# Witchcraft beliefs around the world: An exploratory analysis

**DOI:** 10.1371/journal.pone.0276872

**Published:** 2022-11-23

**Authors:** Boris Gershman

**Affiliations:** Department of Economics, American University, Washington, DC, United States of America; University of Glasgow, UNITED KINGDOM

## Abstract

This paper presents a new global dataset on contemporary witchcraft beliefs and investigates their correlates. Witchcraft beliefs cut across socio-demographic groups but are less widespread among the more educated and economically secure. Country-level variation in the prevalence of witchcraft beliefs is systematically linked to a number of cultural, institutional, psychological, and socioeconomic characteristics. Consistent with their hypothesized function of maintaining order and cohesion in the absence of effective governance mechanisms, witchcraft beliefs are more widespread in countries with weak institutions and correlate positively with conformist culture and in-group bias. Among the documented potential costs of witchcraft beliefs are disrupted social relations, high levels of anxiety, pessimistic worldview, lack of entrepreneurial culture and innovative activity.

## Introduction

*I see witch beliefs as the standardized nightmare of a group, and I believe that the comparative analysis of such nightmares is not merely an antiquarian exercise but one of the keys to the understanding of society.*

Monica Hunter Wilson (1951)

Beliefs in witchcraft, defined as an ability of certain people to intentionally cause harm via supernatural means, have been documented all over the world, both recently and in the distant past [1, 2]. Although extensive research on the subject has greatly contributed to our understanding of witchcraft beliefs, the bulk of available evidence comes from narrowly focused ethnographic case studies and qualitative cross-cultural comparisons. In contrast, formal statistical analyses, particularly at the global scale, have been lacking, in large part due to the paucity of data [3].

This paper presents a new global dataset on contemporary witchcraft beliefs that covers countries and territories representing roughly one half of the world’s adult population. The data reveal that, far from being a remnant of the past limited to small isolated communities, witchcraft beliefs are highly widespread throughout the modern world. At the same time, there are significant differences in their prevalence within and across nations, and we explore this variation at the individual and country levels.

Our individual-level analysis shows that witchcraft beliefs cut across socio-demographic groups and are negatively associated with age, education, and material well-being. Furthermore, witchcraft beliefs are positively correlated with belief in god and religiosity, but affiliation with Christianity (versus Islam) does not make a significant difference.

Guided by the key themes from the literature, our cross-country analysis focuses on the following four issues: 1) the role of witchcraft beliefs in maintaining conformity and self-governance, 2) their relationship to social capital, psychological well-being, and world outlook, 3) the link between witchcraft beliefs, innovation, and economic development, 4) exposure to misfortunes as a factor in sustaining witchcraft beliefs. We examine 60 characteristics and establish the following patterns. First, witchcraft beliefs are substantially more prevalent in countries with weak institutions and low quality of governance. Second, they are strongly positively correlated with measures of cultural conformity and in-group bias. Third, witchcraft beliefs are associated with the erosion of social capital manifested in low levels of trust and other antisocial attitudes and behaviors. Fourth, people in countries with more widespread witchcraft beliefs display lower levels of life satisfaction, diminished sense of control over life and self-efficacy, along with a higher degree of fatalism. Fifth, witchcraft beliefs are negatively related to creative culture and metrics of innovative activity. Sixth, there is a nonlinear, inverted-U relationship between standard metrics of economic development and the prevalence of witchcraft beliefs. Finally, there is mixed evidence on the role of exposure to misfortunes in promoting witchcraft beliefs. These patterns are robust to accounting for continental fixed effects and a number of potentially confounding characteristics, and are generally consistent with existing views on the costs and benefits of witchcraft beliefs in societies.

Our study relies on the working definition of witchcraft introduced above. This definition matches the survey question used to construct the main variables in our analysis, captures the essence of witchcraft beliefs, and is sufficient to build a basic conceptual framework explaining their behavioral consequences. The key idea of this framework is that witchcraft beliefs generate two types of fear, ubiquitous in communities where such beliefs are present: the fear of witchcraft attacks and the fear of witchcraft accusations and ensuing punishment. These fears affect people’s attitudes and behaviors in fundamental ways as they seek to avoid provoking a witch and being labeled as one, which explains both the negative consequences and the social functions of witchcraft beliefs. The former include depleted trust and mutual help, anxiety and paranoid worldview, limited social mobility, avoidance of risks and unorthodox views or actions, disregard for creativity and innovation. On the flip side is the ability of witchcraft-related fears to generate cultural conformity and maintain group-level cohesion under the threat of punishment (in the form of bewitchment or accusation) for transgressing existing norms and challenging the status quo. Thus, our definition of witchcraft is both parsimonious and powerful enough to generate testable predictions, construct relevant survey-based metrics, and conduct an exploratory empirical analysis.

This paper contributes to the vast interdisciplinary literature on witchcraft beliefs, most recently summarized in [2, 3]. More specifically, it advances the emerging quantitative literature relying on observational data and experiments to investigate the present and historical roles of witchcraft beliefs across societies. Several earlier studies focused on social relations. [4] uses survey and ethnographic data to establish a negative association between witchcraft beliefs and several metrics of social capital, including trust, with a focus on regional variation within Sub-Saharan Africa. [5] conducts experiments in the northern Democratic Republic of the Congo and finds support for the adverse causal effect of witchcraft beliefs on trust. [6] shows that witchcraft-like beliefs among the Mosuo in China effectively split the local community into separate networks and hamper inter-group cooperation. Looking at the historical determinants of witchcraft beliefs, [7] links contemporary variation in their prevalence across ethnic and ancestral groups in Sub-Saharan Africa and Latin America, respectively, to the uneven experience of transatlantic slave trade in the past. Witch trials, killings, and witchcraft-related conflicts have also been studied quantitatively, both in the context of contemporary Sub-Saharan Africa [8, 9] and historical Europe [10]. The present study expands the scale of analysis to the global level to pinpoint the key patterns relating to variation in witchcraft beliefs across individuals and countries.

## A global dataset

The data on contemporary witchcraft beliefs come from a sequence of six survey waves conducted by the Pew Research Center (PRC) between 2008 and 2017 in cooperation with professional survey organizations and covering 95 countries and territories around the world. As detailed in Table A.1 of S1 Appendix, 84 of these surveys represent 95% or more of the total adult population in respective countries. In the remaining 11 cases, representativeness rates vary from 70% in Chad to 94% in Afghanistan largely reflecting inaccessibility of certain areas due to armed conflict, political instability, local restrictions, or geographic remoteness (the results reported below are robust to the exclusion of these 11 cases from the sample). All of the interviews were conducted face-to-face with the exception of countries in Western Europe and the U.S., where surveys were implemented via telephone.

Overall, in their design and content the PRC surveys are similar to the “values surveys” (e.g., World Values Survey and European Values Study) and regional “barometers” (e.g., Afrobarometer and Latinobarómetro) widely used across social sciences to measure culture and conduct comparative analyses at the individual and country levels [11]. However, unlike these popular data sources, the PRC surveys were more focused on religious beliefs and, more importantly for the present study, included several questions that can be used to identify witchcraft believers. While the respondents were asked in various forms about the issues of magic, sorcery, and witchcraft, only one relevant question was present in every single survey: “Do you believe in the evil eye, or that certain people can cast curses or spells that cause bad things to happen to someone?” Although the reference to the evil eye belief, representing the fear of supernatural harm caused by envious glances [12], is somewhat confusing, the second part of the question captures precisely the concept of witchcraft adopted above and thus provides a unique way to pinpoint witchcraft believers in the entire merged survey sample. Altogether, the resulting dataset covers more than 140,000 individuals from 95 countries and territories in 5 continents. Over 40% of all survey respondents claimed to believe in witchcraft.

**Fig 1:**
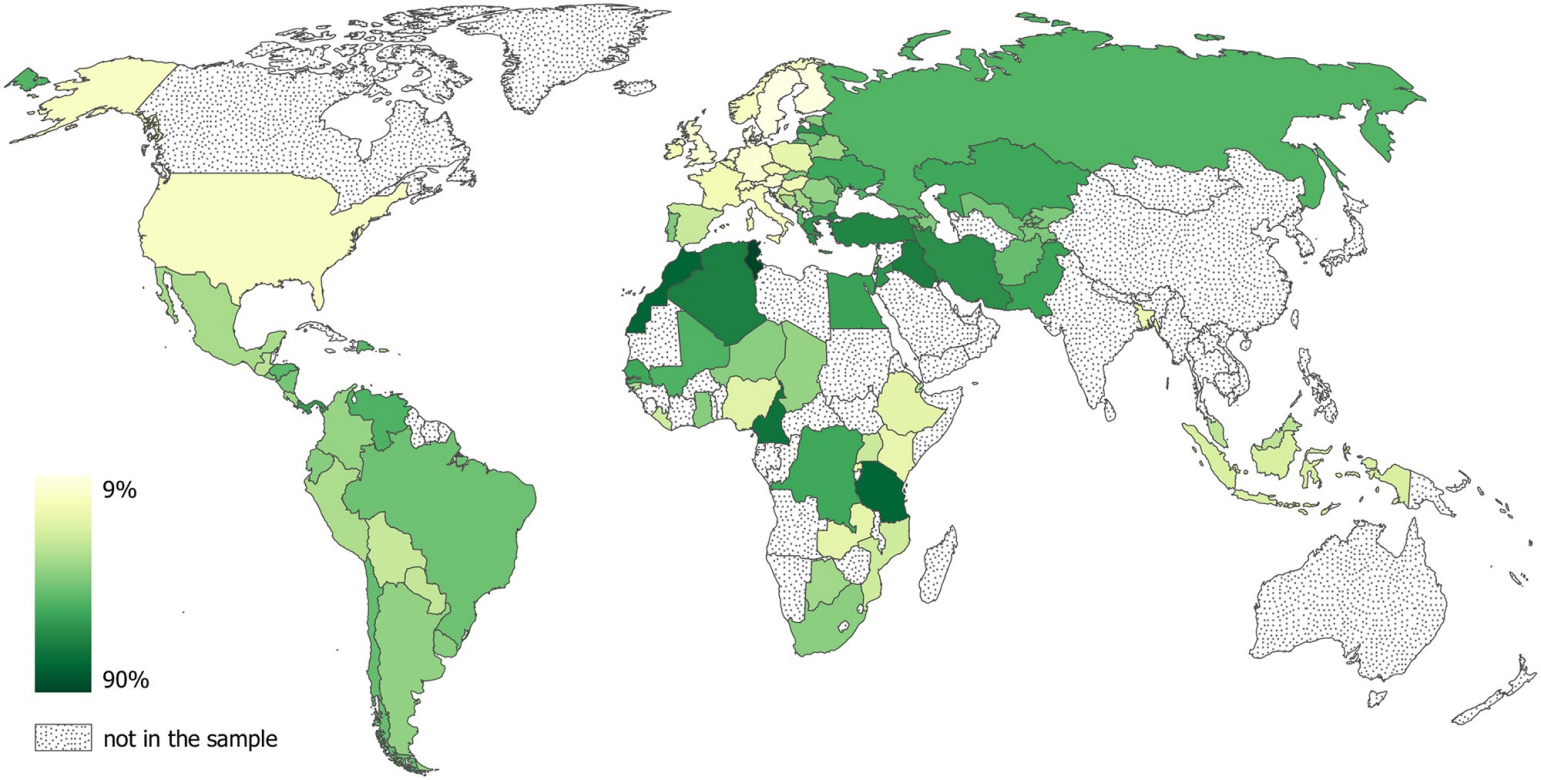
Witchcraft beliefs around the world. Author’s work using country basemap from https://www.naturalearthdata.com.

Fig 1 maps the country-level prevalence of witchcraft beliefs around the world, computed as a fraction of “yes” answers to the above question in the total number of responses. Strikingly, the prevalence rates cover almost the entire possible range varying from 9% in Sweden to 90% in Tunisia, with a mean of 43%. Overall, a simple calculation based on the adult population data yields close to a billion believers in just the 95 countries in the sample, most certainly an undercount due to the sensitivity of the witchcraft question for at least some respondents.

Although the areas covered by the dataset represent roughly a half of the global adult population, Fig 1 reveals several important gaps. Most notably, the surveys did not include China and India, the world’s most populous nations, and generally provide a rather poor coverage of East and Southeast Asia. This, of course, does not mean that witchcraft beliefs are irrelevant in these and other regions not represented in the sample, as the ethnographic literature makes clear, for example, in the cases of India [13], Southeast Asia [14], and Melanesia [15]. These regional gaps in coverage also reflect the focus of the PRC surveys on countries with predominantly Christian and Muslim populations and the resulting lack of representation of other religions. Despite these caveats, our new dataset makes it clear that, first, witchcraft beliefs are a global contemporary phenomenon that is not restricted to just a few selected areas and, second, there is a substantial variation in their prevalence both across and within world regions providing an appealing basis for an exploratory analysis of this paper.

**Fig 2:**
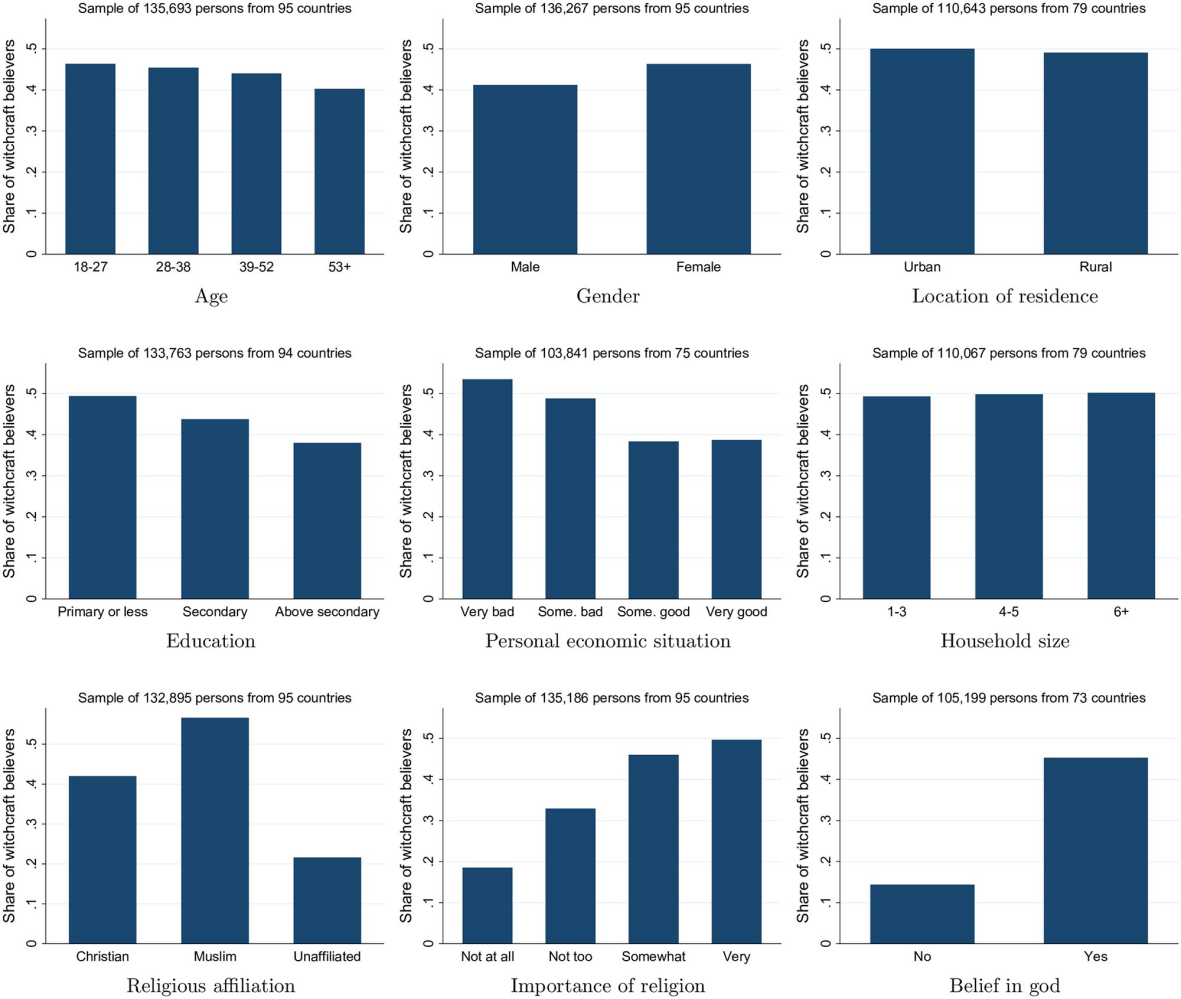
Socio-demographic correlates of witchcraft beliefs. In the sample underlying the religious affiliation panel, about 62% and 27% of respondents identify themselves as Christian and Muslim, respectively, while slightly over 10% are “unaffiliated.” Overall, 95% of witchcraft believers consider themselves either Christian or Muslim.

Before delving into cross-country patterns, we examine the socio-demographic correlates of personal witchcraft beliefs based on the individual-level data in the merged survey sample. Fig 2 shows the “raw” bivariate relationships, based on harmonized variable definitions across survey waves, whereas Table 1 reports estimates from regression models including various characteristics simultaneously while controlling for country fixed effects (and thus capturing relevant nation-level factors). Differences in sample size across the panels of Fig 2 and columns of Table 1 reflect data availability constraints. Most importantly, the personal economic situation question was not asked in Central and Eastern Europe and the U.S., while the urban location and household size variables are missing in the Western Europe wave. Note that, since each country is only covered in one survey wave, it is not feasible to additionally include wave or year fixed effects. For comparison, Table B.2 in S1 Appendix provides estimates for specifications of Table 1 when accounting for just the wave, but not country, fixed effects (these estimates are largely similar to the baseline reported below). Section A of S1 Appendix provides detailed definitions of all variables and Table A.2 in S1 Appendix presents summary statistics.

**Table 1:** Socio-demographic correlates of witchcraft beliefs: Regression estimates.

	(1)	(2)	(3)	(4)	(5)	(6)	(7)	(8)
**Age**	-0.002	-0.006**	-0.008***	-0.006*	-0.006*	-0.008***	-0.008***	-0.007**
	(0.002)	(0.002)	(0.003)	(0.003)	(0.003)	(0.002)	(0.003)	(0.003)
**Gender**: woman	0.048***	0.046***	0.016**	0.011	0.010	0.039***	0.042***	0.011
	(0.008)	(0.009)	(0.007)	(0.008)	(0.008)	(0.008)	(0.008)	(0.007)
**Education**: vs. “primary or less”								
Some or completed secondary		-0.037***	-0.034***	-0.033***	-0.034***	-0.032***	-0.043***	-0.030***
		(0.008)	(0.008)	(0.009)	(0.010)	(0.008)	(0.008)	(0.009)
Above secondary		-0.079***	-0.070***	-0.068***	-0.070***	-0.070***	-0.084***	-0.065***
		(0.013)	(0.014)	(0.015)	(0.016)	(0.012)	(0.012)	(0.015)
**Econ. situation**: vs. “very bad”								
Somewhat bad			-0.034***	-0.033***	-0.033***			-0.033***
			(0.009)	(0.010)	(0.010)			(0.010)
Somewhat good			-0.071***	-0.057***	-0.058***			-0.059***
			(0.010)	(0.010)	(0.010)			(0.011)
Very good			-0.069***	-0.066***	-0.066***			-0.065***
			(0.012)	(0.014)	(0.014)			(0.014)
**Household size**: vs. 1–3								
4–5				0.004	0.004			0.003
				(0.006)	(0.006)			(0.006)
6 and above				0.018**	0.019**			0.018**
				(0.009)	(0.009)			(0.008)
**Urban resident**					0.010			0.013
					(0.009)			(0.009)
**Religion**: vs. Christian								
Muslim						0.014	0.013	-0.009
						(0.024)	(0.024)	(0.029)
Unaffiliated						-0.060***	-0.033**	0.019
						(0.016)	(0.015)	(0.023)
**Imp. of religion**: vs. “not at all”								
Not too important						0.108***		0.058**
						(0.011)		(0.027)
Somewhat important						0.196***		0.107***
						(0.012)		(0.029)
Very important						0.195***		0.107***
						(0.015)		(0.029)
**Belief in god**							0.222***	
							(0.010)	
Observations	135,693	133,244	101,264	75,746	75,746	129,037	101,556	73,849
Countries	95	94	74	58	58	94	73	58

*Notes*. The binary dependent variable is personal belief in witchcraft. Maximum likelihood estimates of marginal effects from probit regressions are reported in all columns. Standard errors clustered by country are shown in parentheses. ***, **, and * denote statistical significance at the 1, 5, and 10 percent level, respectively. Country fixed effects are included in all specifications. Age is measured in tens of years. Data are not weighted. The number of observations and countries for each specification reflects data availability constraints. Table B.1 in S1 Appendix presents (similar) estimates from the linear probability model.

The bivariate patterns and regression estimates yield qualitatively similar conclusions. Witchcraft beliefs are slightly more prevalent among younger people, women, and urban residents in the raw data, although the role of gender is not robust across specifications in Table 1 and the urban location indicator is statistically insignificant in the regression setting. More educated and economically secure individuals are less likely to believe in witchcraft, as are those living in smaller households. Although these correlations are consistent with simple modernization theory, witchcraft beliefs are present across all socio-demographic categories and the group mean differences are relatively mild. The estimates in Table 1 imply that, other things equal, an individual reporting a “very good” personal economic situation is 6–7 percentage points less likely to believe in witchcraft compared to someone in a “very bad” economic situation. Education “above secondary” relative to “primary or less” makes a quantitatively similar difference.

The relationship between witchcraft beliefs and religion is illustrated in the third row of Fig 2. Although the bivariate correlation implies that the prevalence of witchcraft beliefs is higher among Muslims, this pattern is driven by cross-country differences. As shown in columns 6–8 of Table 1, accounting for country fixed effects, that is, effectively comparing Christians and Muslims within countries where they coexist, there is no statistically significant difference in the prevalence of witchcraft beliefs between these two groups. Religiously “unaffiliated” individuals, including atheists and agnostics, are less likely to believe in witchcraft relative to Christians (based on model specifications of columns 6 and 7) and Muslims (based on re-estimating these models after setting Muslims as the reference religious affiliation). Note that the coefficient estimate for the “unaffiliated” changes sign and becomes statistically insignificant in column 8. This happens because, due to missing data, the sample underlying this most demanding specification excludes the entire survey waves for Europe and the U.S., where most of the “unaffiliated” are found. Those who believe in god and consider religion to be an important part of their lives are also more likely to be witchcraft believers. Overall, religious and witchcraft beliefs, both centered on the key role of supernatural powers in life, go hand in hand.

Our analysis thus far shows that witchcraft beliefs are present throughout the world and cut across socio-demographic groups while also revealing certain regularities at the individual level. The following section examines cross-country variation in the prevalence of witchcraft beliefs and its links to cultural, institutional, psychological, and socioeconomic characteristics.

## Cross-country patterns

In the spirit of an exploratory analysis, our main goal is to establish robust patterns of correlation rather than identify causal relationships, the latter being complicated by the challenge of finding quasi-experimental variation in witchcraft beliefs or any other country-level characteristics. To this end, we expand our dataset to include dozens of relevant variables constructed and compiled using a variety of data sources described in section A of S1 Appendix. Note that all variables based on individual-level survey data, including the prevalence of witchcraft beliefs, are aggregated to the country level using appropriate weights provided in the original sources. Although the PRC surveys were conducted at somewhat different points in time between December 2008 and August 2017 (see Table A.1 of S1 Appendix), we consider country-level prevalence rates of witchcraft beliefs to be comparable within the sample: these rates are unlikely to change substantially within a period of several years, given the notorious persistence of religious beliefs and other cultural characteristics [16]. Furthermore, continental fixed effects included in the analysis to a significant degree reflect survey waves that were largely conducted by major geographic region. Summary statistics for all country-level variables are provided in Table A.3 of S1 Appendix.

We formalize the analysis by estimating multivariate linear regression equations, in which witchcraft beliefs appear either on the left- or the right-hand side, depending on the theoretical hypothesis being explored and pre-existing evidence (fixing their position on the same side of the equation regardless of context does not change the qualitative results). For example, when considering the indicators typically viewed in the literature as the social costs, or consequences, of witchcraft beliefs, it is more natural to model the latter as an “independent” variable. In contrast, when exploring the hypothesized determinants of witchcraft beliefs, it is natural to view them as a “dependent” variable. Although this flexible approach makes the presentation and interpretation of results more convenient, it is important to be mindful of the correlational nature of cross-country relationships estimated below.

For consistency, we rely on a similar set of control variables across specifications. First, we include continental fixed effects (for Africa, Americas, Asia, and Europe) making sure that our estimates do not simply reflect differences across world regions. Second, we control for several key geographic characteristics, namely absolute latitude, terrain ruggedness, agricultural suitability of land, and distance to the coastline, all of which have been argued to represent important exogenous determinants of socioeconomic and cultural outcomes [17–19]. Third, in further robustness checks, we additionally account for potentially confounding endogenous characteristics including income per capita, religiosity, historical strength of kinship ties [20], and the quality of institutions. We standardize the main variables of interest to have zero mean and unit standard deviation in relevant samples of countries and present estimation results graphically for their easy visual comparison across model specifications. Section C of S1 Appendix further illustrates selected patterns in the form of scatterplots.

### Institutions and conformity

The idea that witchcraft-related fears enforce cultural conformity and social cohesion goes back to the classical work of Evans-Pritchard on the Azande [21] and Kluckhohn on the Navajo [22]. This “social control” function was shown to play a role in communities across the world, particularly where formal mechanisms of governance and conflict resolution are missing or defunct [23, 24]. It is in such cases that witchcraft beliefs may provide a useful alternative mechanism of maintaining order that benefits societies at the group level [25].

**Fig 3:**
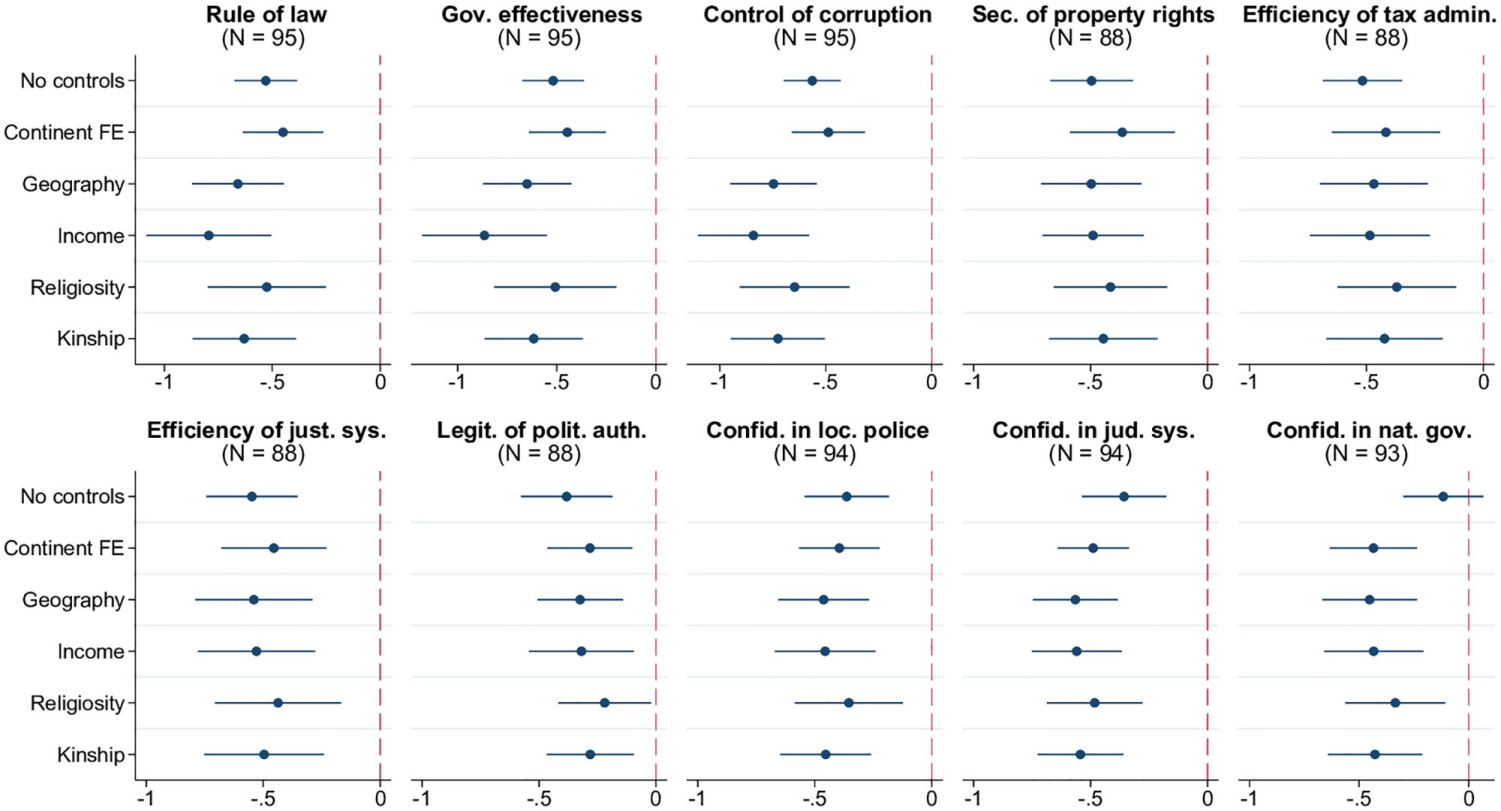
Witchcraft beliefs and institutions. Each panel of the figure presents the results of estimating 6 different models, in which the prevalence of witchcraft beliefs at the country level is regressed on the metric of institutions indicated in the panel title, along with a set of control variables. The latter is defined as follows according to the tickmarks on the vertical axis: 1) none for “No controls”, 2) only continental fixed effects for “Continent FE”, 3) continental fixed effects and baseline geographic controls (absolute latitude, terrain ruggedness, agricultural suitability of land, distance to the coastline) for “Geography.” The remaining 3 models, named “Income,” “Religiosity,” and “Kinship,” include, respectively, real GDP per capita, average religiosity, and kinship intensity index (in addition to continental fixed effects and geographic variables). The round marker represents the point estimate for the coefficient on the respective index of institutions, and the linear segment around each marker is the corresponding 95% confidence interval based on heteroskedasticity-robust standard errors. Confidence intervals that do not cross the reference vertical line at 0 correspond to statistical significance of the respective point estimates at the 5% level. Sample size *N* indicated in parentheses. The key variables are standardized to have zero mean and unit standard deviation in relevant samples.

Fig 3 shows that, indeed, witchcraft beliefs are substantially more widespread in countries with weak formal institutions and low state capacity. The first set of metrics used for this analysis are expert-opinion-based indices of the rule of law, government effectiveness, control of corruption, efficiency of tax administration, legitimacy of political authorities, proper functioning of the justice system, and security of property rights, obtained from the Worldwide Governance Indicators and Institutional Profiles databases. The second set of metrics captures perceptions of institutional quality based on public opinion surveys (namely the Gallup World Poll, hereafter GWP) and delivers the same message: witchcraft beliefs are more prevalent in countries with lower confidence in local police, judicial system, and national government.

This relationship is robust to potentially confounding characteristics, including income per capita, and is quantitatively important: for example, other things equal, a one-standard-deviation increase in the rule-of-law index is associated with an average reduction in the prevalence of witchcraft beliefs by more than 0.5 standard deviations (or 9 percentage points) in models accounting for geography, income, religiosity, and kinship tightness, in addition to continental fixed effects. Given this strong connection, the following analyses incorporate the rule-of-law index as an additional control variable.

**Fig 4:**
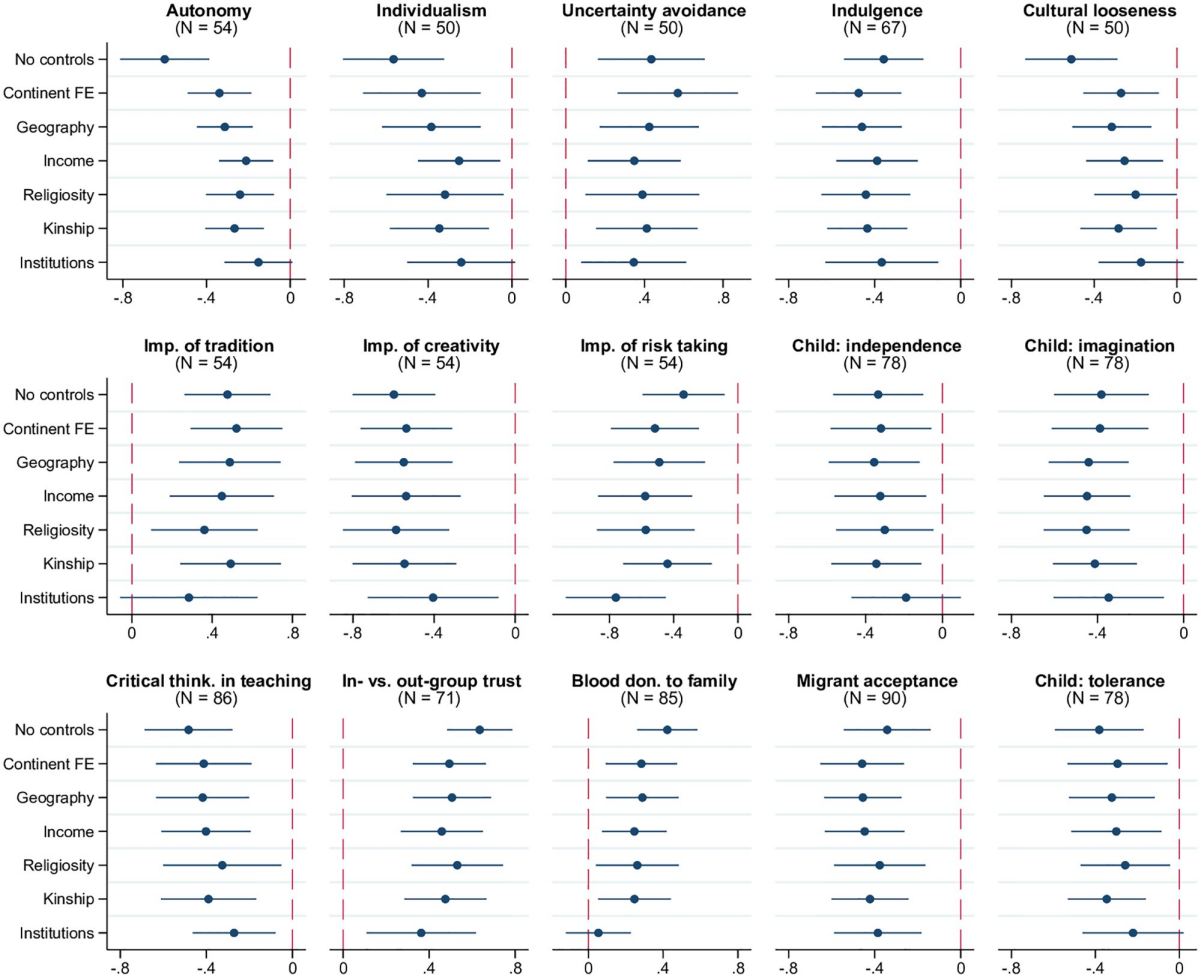
Witchcraft beliefs, conformity, and in-group bias. Each panel of the figure presents the results of estimating 7 different models, in which the metric of conformity indicated in the panel title is regressed on the prevalence of witchcraft beliefs, along with a set of control variables. The latter is defined as follows according to the tickmarks on the vertical axis: 1) none for “No controls”, 2) only continental fixed effects for “Continent FE”, 3) continental fixed effects and baseline geographic controls (absolute latitude, terrain ruggedness, agricultural suitability of land, distance to the coastline) for “Geography.” The remaining 4 models, named “Income,” “Religiosity,” “Kinship,” and “Institutions” include, respectively, real GDP per capita, average religiosity, kinship intensity index, and the rule-of-law index (in addition to continental fixed effects and geographic variables). The round marker represents the point estimate for the coefficient on the prevalence of witchcraft beliefs, and the linear segment around each marker is the respective 95% confidence interval based on heteroskedasticity-robust standard errors. Sample size *N* indicated in parentheses. The key variables are standardized to have zero mean and unit standard deviation in relevant samples.

The main channel through which witchcraft beliefs have been argued to maintain social cohesion is the enforcement of conformity due to expected punishment for norm violation in the form of witchcraft attacks or accusations. The patterns documented in Fig 4 support the close connection between witchcraft beliefs and conformism. The first set of relevant measures includes multiple scales capturing cultural conformity and tightness. The “embeddedness vs. autonomy” scale reflects the extent to which societies view their members as part of a group rather than independent individuals [26]. “Embedded” cultures operate against the disruption of traditional order and value obedience and conformity over creativity and independence. A similar dichotomy is captured by the well-known “individualism vs. collectivism” scale [27]. The first two panels in Fig 4 show that a higher prevalence of witchcraft beliefs is associated with lower degrees of autonomy and individualism. In addition, as illustrated in the next two panels, countries with more widespread witchcraft beliefs score higher on the “uncertainty avoidance” scale and lower on the “indulgence vs. restraint” scale reflecting reliance on rigid social norms, conservative values, and suppression of the basic human drive to enjoy life [27]. Consistent with these results, witchcraft beliefs are also negatively correlated with the cultural looseness index [28] capturing homogeneity of people’s values, norms, and behaviors in society.

The second line of evidence is based on more specific aspects of conformist culture. When asked about issues and character traits they consider valuable, respondents in countries with more widespread witchcraft beliefs are more likely to stress the importance of tradition and downplay the role of creativity and risk taking, based on the data from the World Values Survey (WVS) and the European Values Study (EVS). Similar conformist pattern is seen in the approaches to child socialization, both at home and in school, across societies where witchcraft beliefs are more common: independence and imagination are less frequently mentioned in WVS/EVS as important qualities to cultivate in children, while the prevalent style of instruction, as captured in the 2018 Global Competitiveness Report (GCR), is focused on memorizing and rule-following rather than promoting creative and critical thinking.

The last four panels of Fig 4 examine another dimension of conformity, namely the extent of in-group bias and xenophobic attitudes as captured by four different metrics. First, witchcraft beliefs are positively related to the gap between in- and out-group trust measured, respectively, as average trust across in-groups (family, neighbors, and other acquaintances) and out-groups (newly met individuals and people of another religion and nationality) based on WVS/EVS responses. Second, they are positively related to the share of blood donations to family members [20], although this correlation is sensitive to accounting for institutional quality. Third, in countries with a higher prevalence of witchcraft beliefs, people are less supportive of immigrants living in their country, becoming their neighbors, and marrying into their families, as captured by the lower values of Gallup’s migrant acceptance index. Finally, in-group bias in such societies is cultivated since childhood as shown in the lower importance attached to instilling tolerance and respect for other people in children (WVS/EVS).

The evidence presented so far supports the view that witchcraft beliefs represent a simple mechanism of self-governance and operate to maintain traditional order, promote conformism, and contribute to in-group cohesion. While this constitutes one of the plausible social benefits of witchcraft beliefs, it likely comes with a range of individual and social costs explored in the following sections.

### Social relations, anxiety, and worldview

**Fig 5:**
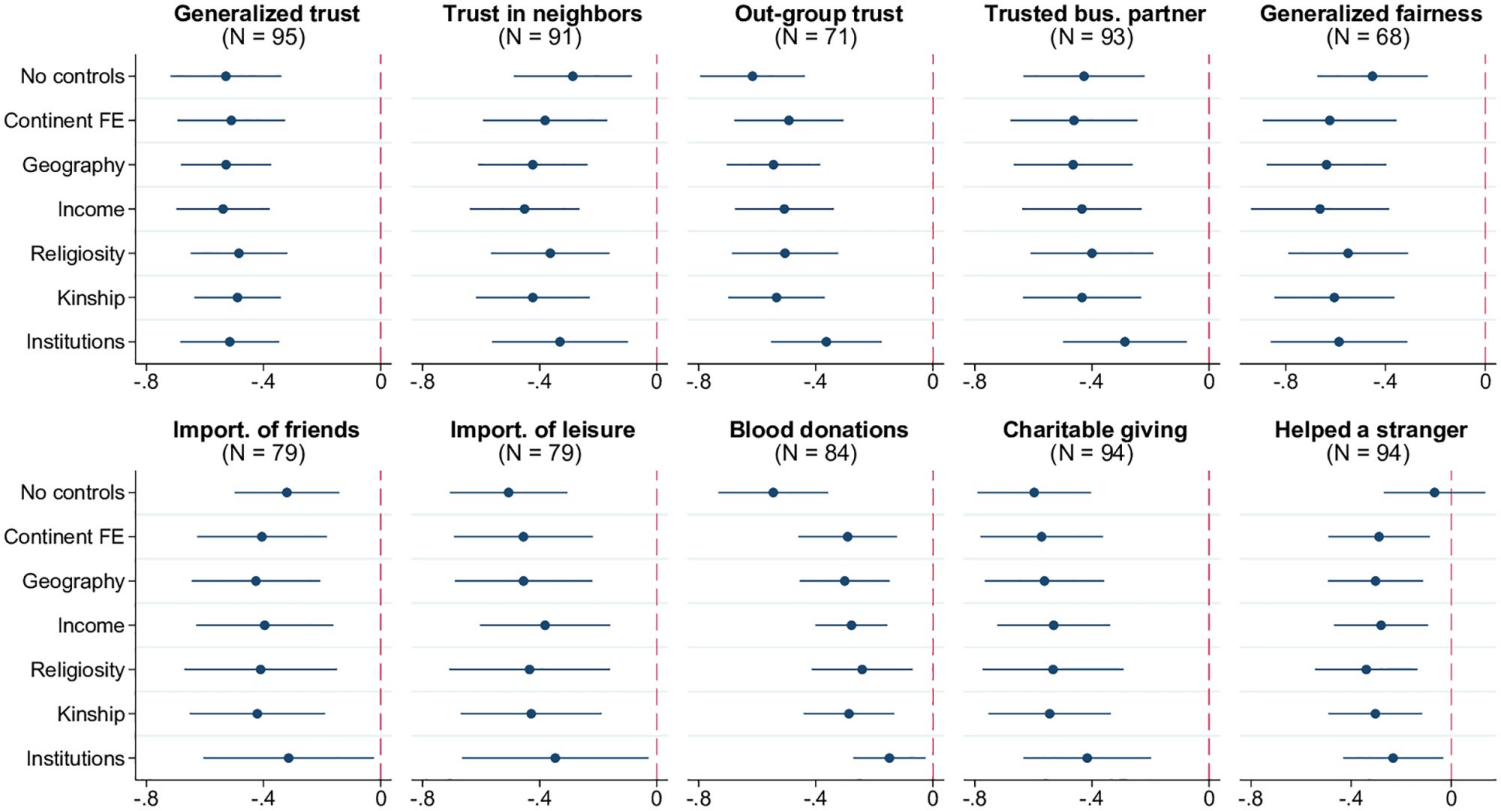
Witchcraft beliefs and ruptured social relations. Each panel of the figure presents the results of estimating 7 different models, in which the metric of social relations indicated in the panel title is regressed on the prevalence of witchcraft beliefs, along with a set of control variables. The latter is defined as follows according to the tickmarks on the vertical axis: 1) none for “No controls”, 2) only continental fixed effects for “Continent FE”, 3) continental fixed effects and baseline geographic controls (absolute latitude, terrain ruggedness, agricultural suitability of land, distance to the coastline) for “Geography.” The remaining 4 models, named “Income,” “Religiosity,” “Kinship,” and “Institutions” include, respectively, real GDP per capita, average religiosity, kinship intensity index, and the rule-of-law index (in addition to continental fixed effects and geographic variables). The round marker represents the point estimate for the coefficient on the prevalence of witchcraft beliefs, and the linear segment around each marker is the respective 95% confidence interval based on heteroskedasticity-robust standard errors. Sample size *N* indicated in parentheses. The key variables are standardized to have zero mean and unit standard deviation in relevant samples.

As argued in previous studies, witchcraft beliefs and related fears are associated with the erosion of social capital including diminished cooperation and mutual help, mistrust, and a general lack of friendly social interactions [4–6, 29]. Consistent with this notion, Fig 5 shows that countries with more widespread witchcraft beliefs are characterized by strained social relations as manifested in lower levels of “generalized” trust, trust in neighbors, out-group trust, and a smaller share of people believing they can find a trusted business partner outside their own family (WVS/EVS; PRC; GWP). Closely related to mistrust is the diminished “generalized fairness,” that is, perception of people as trying to be fair rather than take advantage of others (WVS/EVS). Ruptured community relations are further reflected in lower importance of friends and leisure time in life, as reported in WVS/EVS. Finally, the last three panels in Fig 5 show that prosocial behavior, in addition to attitudes, is also negatively associated with witchcraft beliefs. This includes lower per capita levels of voluntary blood donations to non-family [20] and fewer positive survey responses regarding recent experiences of charitable contributions and helping strangers in need (GWP).

**Fig 6:**
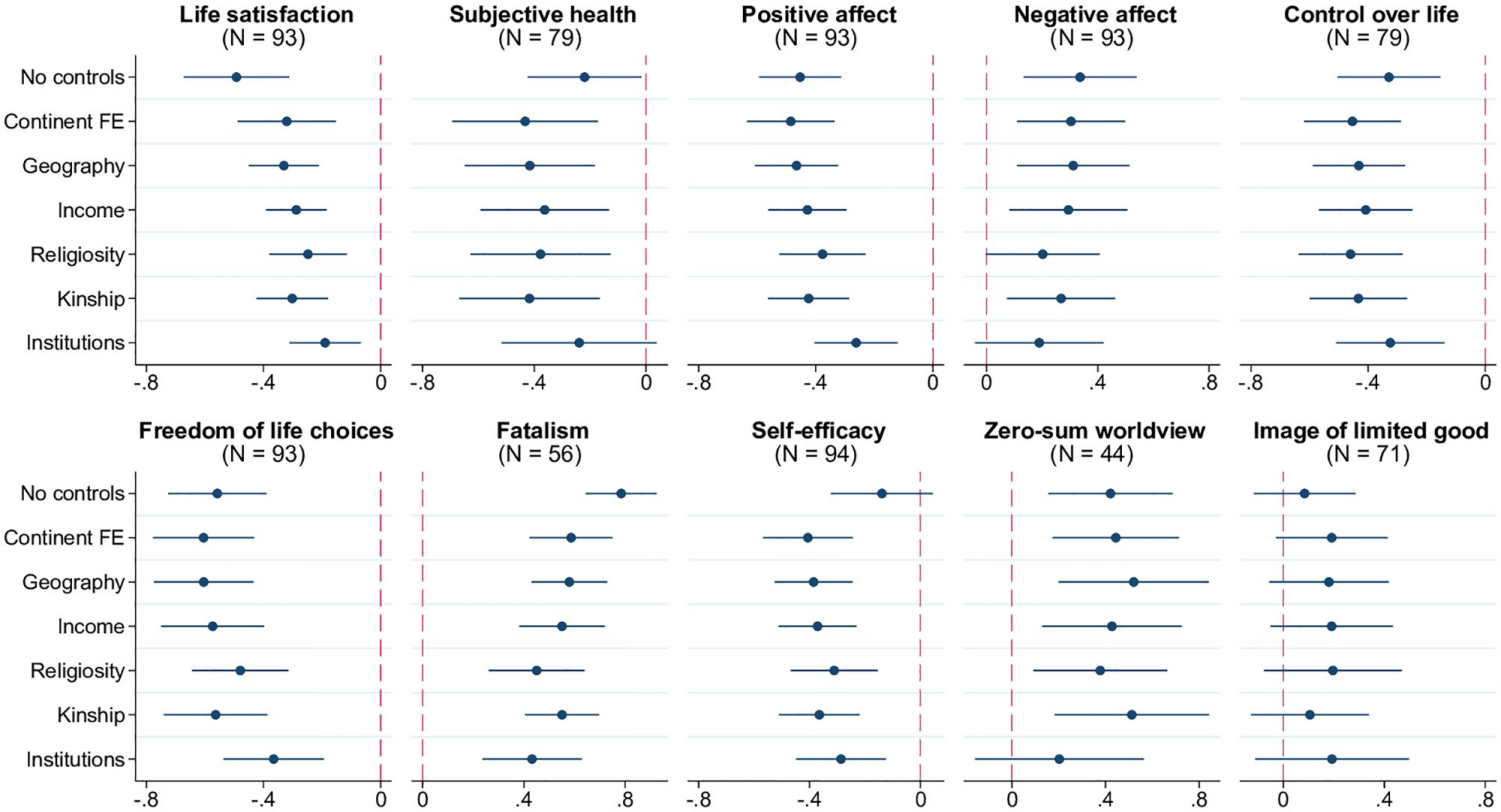
Witchcraft beliefs, anxiety, and worldview. Each panel of the figure presents the results of estimating 7 different models, in which the metric of anxiety or worldview indicated in the panel title is regressed on the prevalence of witchcraft beliefs, along with a set of control variables. The latter is defined as follows according to the tickmarks on the vertical axis: 1) none for “No controls”, 2) only continental fixed effects for “Continent FE”, 3) continental fixed effects and baseline geographic controls (absolute latitude, terrain ruggedness, agricultural suitability of land, distance to the coastline) for “Geography.” The remaining 4 models, named “Income,” “Religiosity,” “Kinship,” and “Institutions” include, respectively, real GDP per capita, average religiosity, kinship intensity index, and the rule-of-law index (in addition to continental fixed effects and geographic variables). The round marker represents the point estimate for the coefficient on the prevalence of witchcraft beliefs, and the linear segment around each marker is the respective 95% confidence interval based on heteroskedasticity-robust standard errors. Sample size *N* indicated in parentheses. The key variables are standardized to have zero mean and unit standard deviation in relevant samples.

The same witchcraft-related fears that disrupt normal social relations have also been argued to drive anxiety and a pessimistic worldview. The stress-inducing impact of witchcraft beliefs is well-documented in both early ethnographic work on the subject and recent studies [22, 30–32]. Psychometric research also found that beliefs in the supernatural, including witchcraft, are generally associated with an external locus of control, that is, attribution of personal outcomes to outside forces such as chance and supernatural powers [33]. Cross-country evidence supports these links. As shown in Fig 6, residents of countries with widespread witchcraft beliefs have lower levels of life satisfaction, based on the 2019 World Happiness Report (WHR), and are more likely to assess the state of their health as poor (WVS/EVS). They also report fewer “positive affect” experiences of happiness, laughter, and enjoyment, and more “negative affect” experiences of worry, sadness, and anger (WHR). Furthermore, there is a very strong relationship between witchcraft beliefs and perceived lack of control over life (WVS/EVS) and inability to freely make life choices (WHR). They are also positively associated with fatalism (PRC) and negatively with self-efficacy, that is people’s belief in their ability to advance in life through own effort and hard work (GWP).

Closely related to fatalism and the lack of personal agency is the zero-sum mindset commonly underlying witchcraft accusations [7]. According to this view, one person’s gain is always someone else’s loss, and witchcraft is seen as a method to achieve individual success at the expense of other community members. [34] proposed a “belief in a zero-sum game” scale to capture such worldview at the country level. As shown in Fig 6, there is a positive relationship between this scale and the prevalence of witchcraft beliefs, although it loses statistical significance when controlling for the quality of institutions. A weaker positive correlation is also observed for the “image of limited good” [35] measure based on the WVS/EVS question asking whether “people can only get rich at the expense of others” or “wealth can grow so there is enough for everyone.”

Although eroded social relations, anxiety, and perceived loss of control over life are all plausible costly consequences of witchcraft beliefs, causality may simultaneously run in the opposite direction. For instance, by hampering cooperation, witchcraft beliefs may aggravate the living conditions of community members and increase the incidence of misfortunes driving mutual accusations. Similarly, by providing an “explanation” for the apparent lack of control over life and poor state of health, witchcraft beliefs may address the basic need for “making sense” of certain life events and coping with adversity and stress. In general, the apparent coexistence of witchcraft beliefs, poor social relations, and pessimistic worldview may be seen as a “cultural package” of mutually reinforcing antisocial beliefs and norms. This stands in sharp contrast to the prosocial cultural package typically associated with religions featuring moralizing high gods [36].

### Innovation and economic development

**Fig 7:**
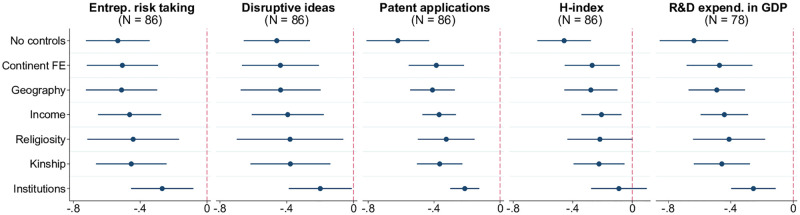
Witchcraft beliefs and innovation. Each panel of the figure presents the results of estimating 7 different models, in which the metric of innovation indicated in the panel title is regressed on the prevalence of witchcraft beliefs, along with a set of control variables. The latter is defined as follows according to the tickmarks on the vertical axis: 1) none for “No controls”, 2) only continental fixed effects for “Continent FE”, 3) continental fixed effects and baseline geographic controls (absolute latitude, terrain ruggedness, agricultural suitability of land, distance to the coastline) for “Geography.” The remaining 4 models, named “Income,” “Religiosity,” “Kinship,” and “Institutions” include, respectively, real GDP per capita, average religiosity, kinship intensity index, and the rule-of-law index (in addition to continental fixed effects and geographic variables). The round marker represents the point estimate for the coefficient on the prevalence of witchcraft beliefs, and the linear segment around each marker is the respective 95% confidence interval based on heteroskedasticity-robust standard errors. Sample size *N* indicated in parentheses. The key variables are standardized to have zero mean and unit standard deviation in relevant samples.

The conformist culture and resistance to change promoted by witchcraft beliefs poses a threat to the process of innovation, a backbone of long-run economic growth. Country-level data from the GCR are consistent with this hypothesis, as shown in Fig 7. Similar to earlier results, there is a strong negative relationship between witchcraft beliefs and expert-opinion-based measures of innovative culture evaluating people’s appetite for taking entrepreneurial risk and the willingness of businesses to embrace disruptive ideas. Beyond attitudes, negative correlations also hold for standard metrics of actual innovative activity including patent applications, scientific publications, and the share of expenditures on research and development in GDP.

**Fig 8:**
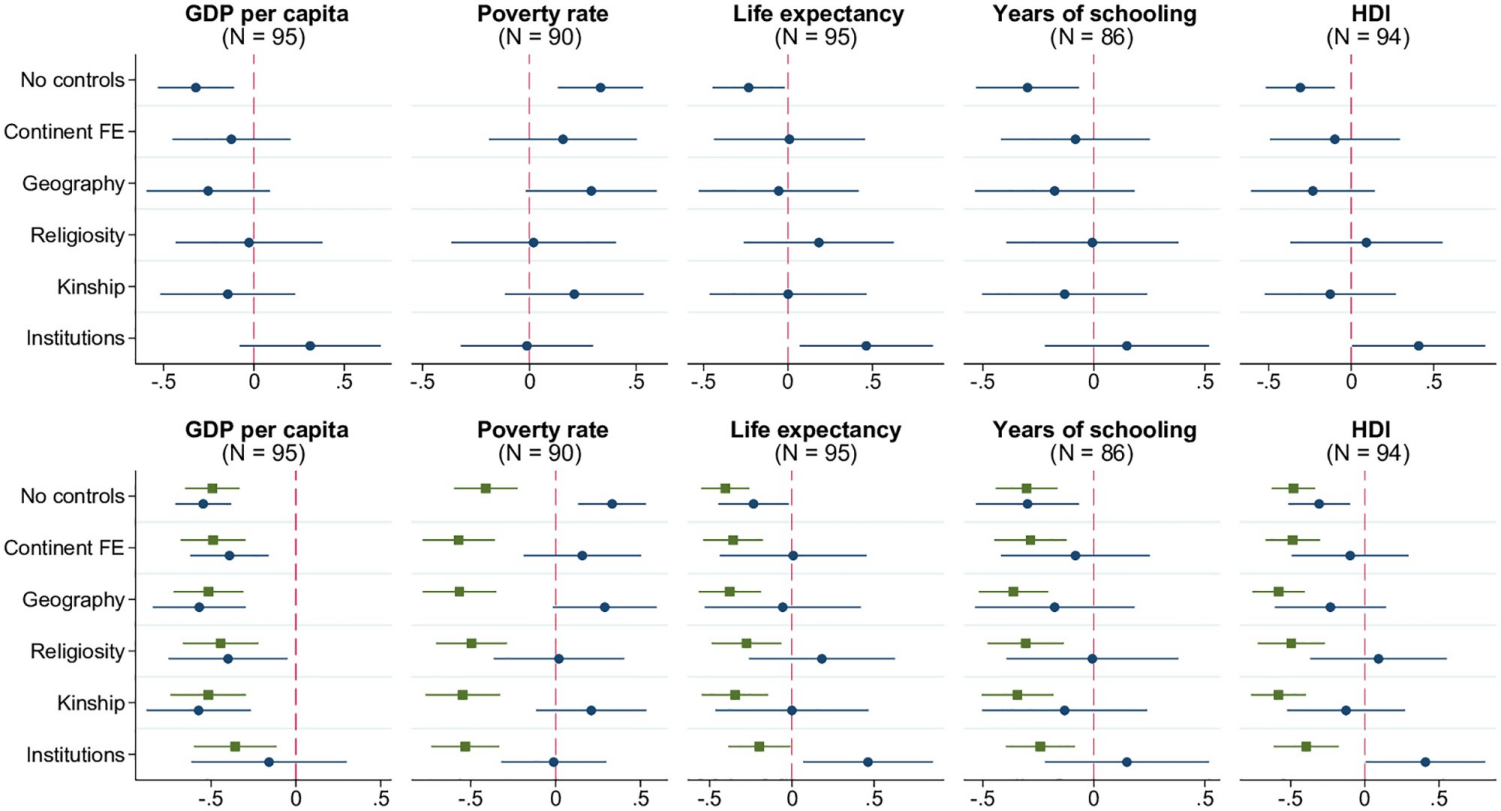
Witchcraft beliefs and development. Each panel in the top row of the figure presents the results of estimating 6 different models, in which the prevalence of witchcraft beliefs at the country level is regressed on the metric of development indicated in the panel title, along with a set of control variables. The latter is defined as follows according to the tickmarks on the vertical axis: 1) none for “No controls”, 2) only continental fixed effects for “Continent FE”, 3) continental fixed effects and baseline geographic controls (absolute latitude, terrain ruggedness, agricultural suitability of land, distance to the coastline) for “Geography.” The remaining 3 models, named “Religiosity,” “Kinship,” and “Institutions” include, respectively, average religiosity, kinship intensity index, and the rule-of-law index (in addition to continental fixed effects and geographic variables). The round marker represents the point estimate for the coefficient on the respective development indicator, and the linear segment around each marker is the corresponding 95% confidence interval based on heteroskedasticity-robust standard errors. Model specifications for the bottom row of the figure are identical, but the right-hand side of the regression equation includes both the linear and quadratic terms for development indicators. The square and round markers and associated confidence intervals in the bottom-row panels correspond to the coefficient estimates on the square and linear terms, respectively. Sample size *N* indicated in parentheses. The key variables are standardized to have zero mean and unit standard deviation in relevant samples.

The interplay between witchcraft beliefs and economic development broadly defined is more complicated. As shown in the top row of Fig 8, there are no robust linear patterns involving standard measures of socioeconomic advancement such as real GDP per capita, poverty rate, life expectancy, mean years of schooling, and the composite human development index capturing income, health, and education (World Development Indicators; Penn World Table; GCR; Human Development Report). The absence of a simple relationship may be explained by the multitude of causal pathways connecting the variables of interest. On the one hand, witchcraft beliefs may inhibit the process of development through various channels described earlier, including the erosion of social capital, promotion of anxiety and the culture of conformity, hampering entrepreneurial spirit and innovation. On the other hand, the rise in living standards and other aspects of development likely affect the prevalence of witchcraft beliefs, and the direction of this impact is a priori ambiguous. According to standard modernization theory, witchcraft beliefs should decline in the process of development due to improved security and health, lower exposure to shocks, spread of education and scientific approach to explaining life events. In contrast, the literature on “modernity of witchcraft,” largely inspired by observations from Sub-Saharan Africa, has argued that some aspects of development, namely rising inequality, globalization, technological change, and migration, may instead revive witchcraft beliefs by disrupting established social order [37].

Interestingly, as shown in the second row of Fig 8, there is a statistically significant *quadratic*, inverted-U-type association between development indicators and witchcraft beliefs. This nonlinearity suggests that, other things equal, countries at an intermediate level of development are characterized by the highest prevalence of witchcraft beliefs. One, admittedly speculative interpretation is that the “modernity” effect dominates at relatively early stages of development but eventually gives way to the “modernization” effect at higher levels of socioeconomic maturity. Such pattern and its tentative interpretation are reminiscent of [9] which found that witchcraft-related concerns and conflict across villages in eastern Sierra Leone were most prevalent in communities where traditional agrarian subsistence economy collided with new market-oriented developments. Specifically, the authors detected an inverted-U relationship between witchcraft salience and market integration measured as the degree of reliance on cash crop production. In their interpretation, communities “caught in the middle” between modern and traditional socioeconomic systems were the most vulnerable to manifestations of witchcraft.

### Misfortunes

Through the ages, the most obvious purpose of witchcraft beliefs has been to provide an ultimate explanation for unfortunate events in people’s lives and thus help with coping. Examples of misfortunes historically and presently attributed to witchcraft include death, disease, weather shocks, crop failure, enslavement, accidents, business problems, joblessness, infertility, and marital issues [1, 7, 8, 38].

**Fig 9:**
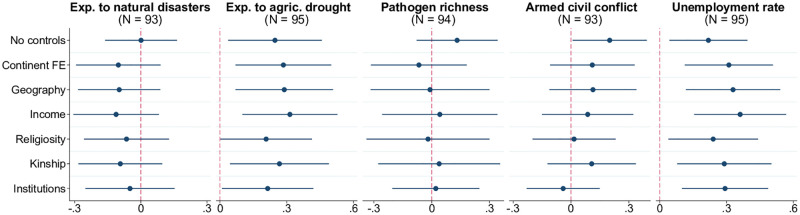
Witchcraft beliefs and exposure to misfortunes. Each panel of the figure presents the results of estimating 7 different models, in which the prevalence of witchcraft beliefs at the country level is regressed on the metric of exposure to misfortune indicated in the panel title, along with a set of control variables. The latter is defined as follows according to the tickmarks on the vertical axis: 1) none for “No controls”, 2) only continental fixed effects for “Continent FE”, 3) continental fixed effects and baseline geographic controls (absolute latitude, terrain ruggedness, agricultural suitability of land, distance to the coastline) for “Geography.” The remaining 4 models, named “Income,” “Religiosity,” “Kinship,” and “Institutions” include, respectively, real GDP per capita, average religiosity, kinship intensity index, and the rule-of-law index (in addition to continental fixed effects and geographic variables). The round marker represents the point estimate for the coefficient on the respective metric of exposure to misfortune, and the linear segment around each marker is the corresponding 95% confidence interval based on heteroskedasticity-robust standard errors. Confidence intervals that do not cross the reference vertical line at 0 correspond to statistical significance of the relevant point estimate at the 5% level. Sample size *N* indicated in parentheses. The key variables are standardized to have zero mean and unit standard deviation in relevant samples.

Fig 9 shows the country-level relationships between the prevalence of witchcraft beliefs and overall exposure to five types of misfortune: natural disasters (earthquakes, storms, floods, droughts, and rising sea levels, based on the 2020 WorldRiskReport), agricultural drought [39], diseases [40], armed civil conflict [41], and unemployment (World Development Indicators). The only two measures showing a robust positive correlation with witchcraft beliefs are exposure to drought, consistent with evidence on crop failures and weather shocks being important triggers of witchcraft accusations, and unemployment rate possibly reflecting the stress-inducing nature of joblessness in the modern world. This mixed overall evidence shows that aggregate shocks need not automatically lead to entrenchment of witchcraft beliefs in societies.

## Conclusion

In her seminal paper quoted in the epigraph [42], Monica Hunter Wilson argued that comparative cross-cultural studies linking witchcraft beliefs to various aspects of societies are essential for understanding the purpose and evolution of these “standardized nightmares.” This paper conducts such a comparative analysis of contemporary witchcraft beliefs at the global scale and reveals their robust association with many individual and country-level characteristics. Consistent with ethnographic evidence on their functional role in maintaining social order, witchcraft beliefs are positively related to conformist culture and are particularly widespread in countries with weak institutions. Witchcraft beliefs are also correlated with exposure to certain shocks such as agricultural drought and unemployment and may provide a coping mechanism for dealing with misfortunes. But these potential functions, or benefits, likely come at a steep cost of destroying the social fabric, contributing to anxiety and economic stagnation.

These multiple facets must be taken into account when considering the implications of witchcraft beliefs in the context of policy interventions, technological, institutional, and cultural changes. One attractive but potentially ineffective avenue is to focus on the large social costs of witchcraft beliefs and attempt drastic changes without thinking through the unintended consequences. An example of such an attempt are various anti-witchcraft laws implemented by colonial and current administrations in developing countries with the goal of preventing witchcraft accusations and persecutions. While reasonable on the surface, such laws have often been disregarded in practice or, even when enforced, raised rather than assuaged witchcraft-related fears since the alleged witches were viewed as being “let loose” and protected by the new laws [1, 43].

Another reasonable but superficial strategy is to focus on education, modernization, and promotion of a scientific worldview as solutions to the issue of witchcraft. While people with higher levels of education and economic security are indeed less likely to believe in witchcraft, these beliefs generally cut across socio-demographic strata. Furthermore, technological development, urbanization, and other aspects of globalization may actually revive rather than alleviate witchcraft concerns by rupturing pre-existing traditional social organization and triggering the conformity-inducing function of witchcraft beliefs and accusations [37]. A focus on instilling an understanding of natural rather than supernatural causes of misfortunes, such as disease or drought, would miss the long-known point about witchcraft [21]: for believers, it provides an ultimate and individualized explanation of misfortune, even when proximate causality mechanisms are well-understood. For instance, a person who accepts mosquito bites as a proximate cause of contracting malaria may, at a deeper level, still attribute a specific disease event to witchcraft.

Finally, an obvious danger is to simply disregard witchcraft beliefs as irrelevant when conducting policy interventions or development projects, that is, fail to take culture into account [44]. Policymakers and researchers may face implementation difficulties, if, for example, a certain project requires mutual trust, cooperation and communal effort, the kind of social capital that is typically lacking in societies with widespread witchcraft beliefs. They may also miss the unintended effects of a project due to witchcraft-related fears such as those that are likely to arise in case of unequal outcomes across community members, for instance, due to selective adoption of a new technology or a novel lending mechanism [45].

Given the goal of minimizing the costs of witchcraft beliefs while minding the functions they may perform in communities, a constructive way to think about policy implementation in this context is the “cultural mismatch” framework recently proposed in [16]. A mismatch happens if, due to its tendency for persistence as a result of intergenerational transmission, the prevalent culture becomes obsolete, that is, offers no clear benefits in the current socioeconomic and institutional environment. Detecting a mismatch is instrumental for policy success since, in its presence, interventions aimed at cultural change are less likely to have undesirable side effects. In the case of witchcraft beliefs, an environment supporting their self-governance and “explanatory” functions is the one of institutional vacuum and vulnerability to external shocks. In such societies, direct attempts to eradicate witchcraft beliefs via laws or curriculum changes are most likely to backfire. On the other hand, in communities where the fundamentals make witchcraft beliefs less relevant, that is, where local institutions effectively maintain order and a social safety net is in place to protect from adverse shocks, policies aimed at reducing the prevalence of witchcraft beliefs, and thus mitigating their costs, are more likely to succeed. The same approach to evaluating local fundamentals may be followed before considering development projects and other interventions in communities with a salient presence of witchcraft beliefs.

## Supporting information

S1 Appendix Supplementary information. This appendix provides further details of the data used in the study, robustness checks, and illustrations.
(PDF)
