# Supplementary material for: Witchcraft beliefs around the world: An exploratory analysis
Source: PLoS One. 2022 Nov 23;17(11):e0276872. doi: 10.1371/journal.pone.0276872 (PMC9683553; doi:10.1371/journal.pone.0276872)
Supplement: S1 Appendix — This appendix provides further details of the data used in the study, robustness checks, and illustrations. (PDF) [file pone.0276872.s001.pdf]

S1 Appendix
Supplementary information

"Witchcraft beliefs around the world: an exploratory analysis"
by Boris Gershman

A Data details

Table A.1: Pew Research Center surveys

Country	Sample size	Representativeness	Excluded areas and/or residents
**1. Tolerance and Tension: Islam and Christianity in Sub-Saharan Africa (12/2008–4/2009)**			
Botswana	1,002	100%	
Cameroon	1,503	100%	
Chad	1,503	70%	Borkou-Ennedi-Tibesti (sparsely populated and unsafe), Mandoul, Moyen-Chari, Ouaddai, Salamat and Wadi Fira (unstable)
D. R. of the Congo	1,519	80%	Inaccessible and unstable areas, some conflict areas along the border with Rwanda
Djibouti	1,500	100%	
Ethiopia	1,500	100%	
Ghana	1,500	100%	
Guinea-Bissau	1,000	100%	
Kenya	1,500	100%	
Liberia	1,500	100%	
Mali	1,000	100%	
Mozambique	1,500	100%	
Nigeria	1,516	100%	
Rwanda	1,000	100%	
Senegal	1,000	100%	
South Africa	1,504	100%	
Tanzania	1,504	100%	
Uganda	1,040	100%	
Zambia	1,000	100%	
Total	25,091		
**2. Religion and Public Life Survey B (8/2009)**			
U.S.A.	2,003	100%	Non-continental U.S.

3. The World's Muslims (10/2011–11/2012)

Afghanistan	1,509	94%	Nomadic populations
Albania	788	98%	Some difficult-to-reach areas
Algeria	1,181	75%	Western region (due to an administrative error)
Azerbaijan	996	85%	Upper Karabakh, Nakhchivan, Kalbacar-Lacin
Bangladesh	1,918	100%	
Egypt	1,798	98%	Five sparsely populated frontier provinces
Indonesia	1,880	87%	Papua and other remote sparsely populated areas
Iran	1,519	100%	
Iraq	1,416	100%	
Jordan	966	100%	
Kyrgyzstan	1,292	100%	
Lebanon	551	98%	Areas of Beirut controlled by a militia group, a few villages near the border with Israel
Malaysia	1,244	100%	
Morocco	1,472	100%	
Niger	946	97%	Agadez
Pakistan	1,450	82%	Federally Administered Tribal Areas, Gilgit-Baltistan, Azad Jammu and Kashmir (security reasons), unstable areas in Khyber Pakhtunkhwa and Balochistan
Palestine	994	95%	Bedouins, some communities near Israeli settlements (due to military restrictions)
Tajikistan	1,453	99%	
Tunisia	1,450	100%	
Turkey	1,485	100%	
Uzbekistan	965	99%	
Total	27,273		

4. Religion in Latin America (10/2013–2/2014)

Argentina	1,512	99%	Tierra del Fuego, inaccessible or sparsely populated areas, villages with fewer than 400 people
Bolivia	1,503	90%	Villages with fewer than 110 people
Brazil	2,000	97%	Remote areas in the Amazon rainforest and interior parts of the Amazonian states
Chile	1,504	99%	Remote areas in the Atacama desert, mountains, on islands and in the far South
Colombia	1,508	97%	Remote areas in the Amazon rainforest and San Andrés island
Costa Rica	1,500	99%	Gated communities and multi-story residential buildings
Dominican Rep.	1,699	100%	

Ecuador	1,850	98%	Remote areas in the Galápagos and non-delimited areas between provinces
El Salvador	1,500	100%	
Guatemala	1,500	98%	Gated communities and multi-story residential buildings
Honduras	1,500	98%	Bay Islands, small urban populations of five departments, gated communities and multi-story residential buildings
Mexico	2,000	100%	
Nicaragua	1,500	99%	Gated communities and multi-story residential buildings
Panama	1,500	100%	
Paraguay	1,504	100%	
Peru	1,500	99%	
Puerto Rico	1,700	100%	
Uruguay	1,506	100%	
Venezuela	1,540	95%	Delta Amacuro, Amazonas, Dependecias Federales, 183 inaccessible (unsafe) parishes
Total	30,326		

5. Religion and Social Life in Central and Eastern Europe (6/2015–7/2016)

Armenia	1,523	100%	
Belarus	1,513	100%	
Bosnia	1,561	99.7%	Some inaccessible remote areas
Bulgaria	1,619	100%	
Croatia	1,616	97.5%	Smallest islands and some sparsely populated rural areas
Czech Republic	1,490	100%	
Estonia	1,689	100%	
Georgia	1,533		Abkhazia and South Ossetia
Greece	1,465	93%	Small islands
Hungary	1,483	99%	Some remote areas
Kazakhstan	1,692	100%	
Latvia	1,649	100%	
Lithuania	1,572	99%	Peripheral farms
Moldova	1,841	100%	
Poland	1,484	100%	
Romania	1,361	98.5%	Danube Delta
Russia	2,471	100%	
Serbia	1,574	99.5%	Some remote sparsely populated areas
Ukraine	2,409		Donetsk and Luhansk regions, Crimea
Total	31,545		

6. Being Christian in Western Europe (4/2017–8/2017)

Austria	1,791	99%	People without cell or landline phones

Belgium	1,500	100%	
Denmark	1,493	99%	People without cell or landline phones
Finland	1,498	100%	
France	1,788	99%	People without cell or landline phones
Germany	2,211	100%	
Ireland	1,499	99%	People without cell or landline phones
Italy	1,804	97%	People without cell or landline phones
Netherlands	1,497	100%	
Norway	1,498	98%	People without cell or landline phones
Portugal	1,501	98%	People without cell or landline phones
Slovakia	1,497	96%	People without cell or landline phones
Spain	1,499	99%	People without cell or landline phones
Sweden	1,493	100%	
Switzerland	1,686	99%	People without cell or landline phones
United Kingdom	1,841	100%	
Total	26,096		

Notes. Representativeness rates reported for the adult population (age 18 or above). The Thailand survey is excluded since it only represents adult Muslims in five southern provinces. The Kosovo survey is excluded due to unavailability of most variables used in the cross-country analysis separately for Kosovo. The surveys in Georgia and Ukraine are representative of 100% of the adult population in covered regions (countrywide numbers are unavailable). *Source*: survey documentation provided by the Pew Research Center.

Table A.2: Summary statistics: individual-level analysis

Variable	Mean	St. dev.	Min	Max	Obs.
Belief in witchcraft, binary	.438	.496	0	1	136,267
Age	42.3	16.9	18	96	135,693
Gender (woman), binary	.52	.5	0	1	136,267
Urban location, binary	.593	.491	0	1	110,643
Belief in god, binary	.861	.346	0	1	105,199
**Education, categories**					133,763
Completed primary or less	.239	.427	0	1	
Some or completed secondary	.474	.499	0	1	
Above secondary	.287	.452	0	1	
**Economic situation, categories**					103,841
Very bad	.111	.314	0	1	
Somewhat bad	.218	.413	0	1	
Somewhat good	.549	.498	0	1	
Very good	.122	.328	0	1	
**Household size, categories**					110,067
1–3	.488	.5	0	1	
4–5	.293	.455	0	1	
6 and above	.219	.414	0	1	
**Religious affiliation, categories**					132,895
Christian	.622	.485	0	1	
Muslim	.273	.445	0	1	
Unaffiliated	.105	.306	0	1	
**Importance of religion, categories**					135,186
Not at all important	.095	.293	0	1	
Not too important	.118	.322	0	1	
Somewhat important	.244	.43	0	1	
Very important	.543	.498	0	1	

Notes. Summary statistics are shown for the sample of people who gave a “yes” or “no” response to the witchcraft question. In addition to missing data for some respondents, several questions were not asked in certain survey waves. Specifically, the personal economic situation question was not asked in Central and Eastern Europe and the U.S., the urban location and household size variables are missing in the Western Europe wave, and the belief in god question is phrased differently and missing in the World’s Muslims and the U.S. surveys, respectively.

Table A.3: Summary statistics: country-level analysis

Variable	Mean	St. dev.	Min	Max	Obs.	Source
Witchcraft beliefs	.43	.18	.089	.9	95	Pew Research Center
**Continent indicators**						
Africa	.25	.44	0	1	95	
Americas	.21	.41	0	1	95	
Asia	.19	.39	0	1	95	
Europe	.35	.48	0	1	95	
**Other control variables**						
Absolute latitude	31	18	.53	64	95	Nunn and Puga (2012)
Terrain ruggedness	1.3	1.1	.037	5.3	95	Nunn and Puga (2012)
Agricultural suitability	1,266	651	5.1	2,743	95	Galor and Özak (2016)
Distance to the coast	.4	.45	.012	2.2	95	Nunn and Puga (2012)
Religiosity	3.3	.62	1.8	4	95	Pew Research Center
Kinship intensity	-.25	.99	-1.6	1.5	95	Schultz et al. (2019)
**Institutions and conformity**						
Rule of law	-.055	1	-1.9	2	95	Worldwide Governance Indicators
Government effectiveness	.032	.92	-1.6	2.1	95	Worldwide Governance Indicators
Control of corruption	-.072	.99	-1.5	2.3	95	Worldwide Governance Indicators
Security of property rights	2.5	.91	.5	4	88	Institutional Profiles database
Efficiency of tax administration	2.6	.88	0	4	88	Institutional Profiles database
Efficiency of justice system	2.4	.75	1	4	88	Institutional Profiles database
Legitimacy of political authorities	2.7	.68	1.3	4	88	Institutional Profiles database
Confidence in local police	.62	.15	.3	.94	94	Gallup World Poll
Confidence in judicial system	.47	.17	.14	.9	94	Gallup World Poll
Confidence in national government	.47	.17	.18	.97	93	Gallup World Poll
Autonomy vs. embeddedness	.13	.84	-1.6	1.6	54	Schwartz (2014)
Individualism vs. collectivism	45	24	6	91	50	Hofstede et al. (2010)
Uncertainty avoidance	72	21	23	112	50	Hofstede et al. (2010)
Indulgence vs. restraint	45	24	0	100	67	Hofstede et al. (2010)
Cultural looseness	54	27	0	120	50	Uz (2015)
Importance of tradition	.51	.33	-.22	1.2	54	WVS/EVS
Importance of creativity	.22	.3	-.64	.88	54	WVS/EVS
Importance of risk taking	-.81	.3	-1.4	-.24	54	WVS/EVS
Child qualities: independence	.43	.14	.21	.81	78	WVS/EVS
Child qualities: imagination	.18	.072	.04	.38	78	WVS/EVS
Critical thinking in teaching	3.5	.84	2.2	5.7	86	Global Competitiveness Report
In- vs. out-group trust	1	.24	.61	1.7	71	WVS/EVS
Share of blood donations to family	.34	.35	0	.97	85	Schultz et al. (2019)
Child qualities: tolerance	.66	.097	.4	.87	78	WVS/EVS
Migrant acceptance index	5.2	1.8	1.7	8.2	90	Gallup World poll

**Social relations, anxiety, and worldview**						
Generalized trust	.25	.14	.043	.69	95	Multiple
Trust in neighbors	3.3	.18	3	3.7	91	WVS/EVS
Out-group trust	2.2	.31	1.6	2.9	71	WVS/EVS
Trusted business partner	.49	.12	.23	.82	93	Gallup World Poll
Generalized fairness	5.6	.79	4.1	8	68	WVS/EVS
Importance of friends	3.3	.22	2.6	3.7	79	WVS/EVS
Importance of leisure	3.1	.23	2.5	3.5	79	WVS/EVS
Blood donations to non-family	16	17	.16	57	84	Schultz et al. (2019)
Recent charitable donation	.27	.15	.044	.7	94	Gallup World Poll
Helped a stranger recently	.48	.098	.3	.78	94	Gallup World Poll
Life satisfaction	5.5	1	3.6	7.6	93	World Happiness Report
Subjective state of health	3.8	.25	3.2	4.3	79	WVS/EVS
Positive affect	.71	.1	.51	.87	93	World Happiness Report
Negative affect	.26	.069	.14	.51	93	World Happiness Report
Locus of control	6.9	.61	5.8	8.3	79	WVS/EVS
Freedom of life choices	.72	.13	.45	.95	93	World Happiness Report
Fatalism	.68	.23	.25	.98	56	Pew Research Center
Self-efficacy	.76	.16	.32	.95	94	Gallup World Poll
Zero-sum worldview	3.6	.39	2.4	4.3	44	Różycka-Tran et al. (2015; 2018; 2019)
Image of limited good	4.7	.59	3.5	6.1	71	WVS/EVS
**Innovation and economic development**						
Entrepreneurial risk taking	50	9.5	30	79	86	Global Competitiveness Report
Embracing disruptive ideas	3.6	.58	2.6	5.7	86	Global Competitiveness Report
Patent applications	27	33	0	100	86	Global Competitiveness Report
H-index	78	13	51	100	86	Global Competitiveness Report
R&D expenditures in GDP	.9	.86	.01	3.3	78	Global Competitiveness Report
Log of real GDP per capita	9.4	1.1	6.9	11	95	Multiple
Log of poverty rate	1.6	2.1	-3.2	4.5	90	World Development Indicators
Life expectancy	72	7.8	52	83	95	World Development Indicators
Mean years of schooling	9.3	3	1.9	14	86	Global Competitiveness Report
Human development index	.73	.15	.36	.95	94	Human Development Report
**Exposure to misfortunes**						
Exposure to natural disasters	15	7.1	3.7	43	93	WorldRiskReport
Exposure to agricultural drought	.93	.44	.042	2.1	95	Meza et al. (2020)
Pathogen richness	208	13	187	248	94	Fincher and Thornhill (2008)
Armed civil conflict	.024	.031	0	.14	93	Arbatlı et al. (2020)
Unemployment rate	8.2	5.2	.85	26	95	World Development Indicators

Notes. WVS and EVS stand for World Values Survey and European Values Study, respectively. Multiple sources for generalized trust are the Pew Research Center, WVS, EVS, and the Gallup World Poll. Multiple sources for the log of real GDP per capita are the World Development Indicators and the Penn World Table 10.0 (for Venezuela only). The cross-country regression analysis uses standardized versions of all variables with zero mean and unit standard deviation in relevant samples. See the detailed definitions below.

Definitions of variables used in the analysis

Witchcraft beliefs

Personal belief in witchcraft. A dummy variable coding "yes" (1) and "no" (0) answers to the following question: "Do you believe in the evil eye, or that certain people can cast curses or spells that cause bad things to happen to someone?" *Source*: Pew Research Center surveys.

Prevalence of witchcraft beliefs at the country level. The fraction of respondents who claim to believe "in the evil eye, or that certain people can cast curses or spells that cause bad things to happen to someone" relative to the total number of respondents. Computed at the country level using individual-level survey weights provided for aggregation purpose. *Source*: Pew Research Center surveys.

Socio-demographic characteristics

All socio-demographic characteristics of respondents are constructed and harmonized based on the original surveys listed in table A.1.

Age. Age of respondent in tens of years.

Gender. A dummy variable equal to 1 (0), if female (male).

Location of residence. A dummy variable equal to 1 (0) for urban (rural) locations.

Education. A categorical variable classifying the data on self-reported educational attainment into three categories: primary or less, secondary, above secondary.

Personal economic situation. A categorical variable reflecting respondents' assessment of their personal economic situation on the following scale: very bad, somewhat bad, somewhat good, very good.

Household size. A categorical variable capturing self-reported household size: 1–3 people, 4–5, and 6 or more.

Religious affiliation. A categorical variable capturing religious affiliation or its absence: Christian, Muslim, unaffiliated (including agnostics and atheists). About 0.5% of respondents representing all other religions are excluded from the sample when using this variable.

Importance of religion. A categorical variable capturing self-reported importance of religion in life: not at all important, not too important, somewhat important, very important.

Belief in god. A dummy variable equal to 1, if the respondent claims to believe in god, and 0, if not.

Baseline control variables

Continental fixed effects. A set of dummy variables indicating the belonging of a given country to one of the following world regions (total number of countries indicated in parentheses): Africa (24), Americas (20), Asia (18), Europe (33).

Absolute latitude. Absolute latitude of the country centroid. *Source*: Nunn and Puga (2012).

Terrain ruggedness. Mean terrain ruggedness index. *Source*: Nunn and Puga (2012).

Distance to the coast. Average distance (in thousands of kilometers) to the nearest ice-free coast. *Source*: Nunn and Puga (2012).

Agricultural suitability of land. Caloric suitability index capturing average potential agricultural output (measured in calories) based on crops that were available for cultivation in the post-1500CE era. *Source*: `https://ozak.github.io/Caloric-Suitability-Index/`, based on Galor and Özak (2016).

Religiosity. Country-level average religiosity based on individual-level data on the importance of religion in life. *Source*: own calculations based on the Pew Research Center surveys.

Kinship ties. Kinship intensity index based on anthropological reports and combining information on five sub-indicators capturing key dimensions of kin-based organization: cousin marriage preference, polygamy, co-residence of extended families, lineage organization, community organization. *Source*: Schulz et al. (2019).

Institutions and conformity

Rule of law. Measures the extent to which agents have confidence in and abide by the rules of society, and in particular the quality of contract enforcement, property rights, the police, and the courts, as well as the likelihood of crime and violence; average across 2008–2017. *Source*: Worldwide Governance Indicators (2020).

Government effectiveness. Measures the quality of public services, the quality of the civil service and the degree of its independence from political pressures, the quality of policy formulation and implementation, and the credibility of the government's commitment to such policies; average across 2008–2017. *Source*: Worldwide Governance Indicators (2020).

Control of corruption. Measures the extent to which public power is exercised for private gain, including both petty and grand forms of corruption, as well as "capture" of the state by elites and private interests; average across 2008–2017. *Source*: Worldwide Governance Indicators (2020).

Security of property rights. Captures the efficiency of legal means to protect property rights in the event of conflict between private stakeholders, the extent of arbitrary pressure exerted on private property by the state, state's compensation for expropriation of land and means of production. *Source*: Institutional Profiles Database (2012).

Efficiency of the tax administration. Captures the efficiency of collecting corporate and household income taxes, the ability to collect taxes across the entire state territory and limit tax evasion. *Source*: Institutional Profiles Database (2012).

Functioning of the justice system. Captures the degree of judicial independence from the state, enforcement of judicial decisions, timeliness of judicial decisions, and equal treatment of citizens and foreigners before the law. *Source*: Institutional Profiles Database (2012).

Legitimacy of political authorities. Captures the strength of political legitimacy stemming from the ability to ensure economic and social benefits, as well as a sense of national pride for large sections of the population. *Source*: Institutional Profiles Database (2012).

Confidence in local police, judicial system and courts, national government. The share of survey respondents expressing confidence in respective institutions; averages of the available data up to 2020. *Source*: own calculations based on the Gallup World Poll data.

Autonomy vs. embeddedness. A scale capturing the extent to which people are autonomous rather than embedded in their groups. Calculated as the difference between the average of "affective" and "intellectual" autonomy scores and embeddedness score. Autonomous cultures "encourage people to cultivate and express their own preferences, feelings, ideas, and abilities, and to find meaning in their own uniqueness." Intellectual autonomy "encourages individuals to pursue their own ideas and intellectual directions independently. Examples of important values in such cultures include broadmindedness, curiosity, and creativity. Affective autonomy encourages individuals to pursue arousing, affectively positive personal experience. Important values include pleasure, exciting life, and varied life." Embedded cultures "treat people as entities embedded in the collectivity. Meaning in life is expected to come largely through in-group social relationships, through identifying with the group, participating in its shared way of life, and striving toward its shared goals. Embedded cultures emphasize maintaining the status quo and restraining actions that might disrupt in-group solidarity or the traditional order. Important values in such cultures are social order, respect for tradition, security, obedience, and wisdom." *Source*: Schwartz (2014), data downloaded at `http://dx.doi.org/10.13140/RG.2.1.3313.3040`.

Individualism vs. collectivism. A scale capturing individualistic societies as opposed to collectivist. Individualism "can be defined as a preference for a loosely-knit social framework in which individuals are expected to take care of only themselves and their immediate families." Collectivism "represents a preference for a tightly-knit framework in society in which individuals can expect their relatives or members of a particular ingroup to look after them in exchange for unquestioning loyalty." *Source*: Hofstede et al. (2010).

Uncertainty avoidance. A scale expressing "the degree to which the members of a society feel uncomfortable with uncertainty and ambiguity." Societies with strong uncertainty avoidance "maintain rigid codes of belief and behaviour, and are intolerant of unorthodox behaviour and ideas." *Source*: Hofstede et al. (2010).

Indulgence vs. restraint. "Indulgence stands for a society that allows relatively free gratification of basic and natural human drives related to enjoying life and having fun. Restraint stands for a society that suppresses gratification of needs and regulates it by means of strict social norms." *Source*: Hofstede et al. (2010).

Cultural looseness. An index constructed based on standard deviations of responses in WVS/EVS pertaining to questions about the roles of work, family, and religion. *Source*: Uz (2015).

Importance of tradition, creativity, risk-taking. These measures are based on Schwartz's human values module of the WVS/EVS. Respondents rate on a six-point scale how much they believe a person described as follows is like them: 1) "Tradition is important to this person; to follow the customs handed down by one's religion or family;" 2) "It is important to this person to think up new ideas and be creative; to do things one's own way;" 3) "Adventure and taking risks are important to this person; to have an exciting life." Following Schwartz's recommendation, responses are adjusted by subtracting the mean answers a

respondent gave to all human values questions; averages across available years 1981–2020. *Source*: own calculations based on WVS/EVS.

Child qualities: independence, imagination, tolerance and respect for other people. Fraction of respondents in the World Values Survey (WVS) or the European Values Study (EVS) indicating respective trait as an important quality to instill in children; average across available years 1981–2020. Note that the survey question prompts the respondents to choose up to 5 such important qualities; "incorrect" responses listing more than 5 qualities were dropped for consistency and surveys with more than 20% of such "faulty" responses were fully excluded. *Source*: own calculations based on WVS/EVS.

Critical thinking in teaching. Based on the following survey question: "In your country, how do you assess the style of teaching?" Measured on a 1–7 scale, where 1 corresponds to "frontal, teacher based, and focused on memorizing" and 7 corresponds to "encourages creative and critical individual thinking." Question originally asked in the World Economic Forum, Executive Opinion Survey; 2017–2018 weighted average or most recent period available. *Source*: World Economic Forum, Global Competitiveness Report (2018).

In- vs. out-group trust. Based on the WVS/EVS trust questions posed in the following way: "I'd like to ask you how much you trust people from various groups. Could you tell me for each whether you trust people from this group completely, somewhat, not very much or not at all?" (responses are numerically coded from 4 to 1, respectively). The groups are (i) family, (ii) neighbors, (iii) people the respondent knows personally, (iv) people met for the first time, (v) people of another religion, and (vi) people of another nationality. The final measure is constructed by taking the difference between the average responses to the first three questions (in-group trust) and the last three questions (out-group trust); average across available years 1981–2020. *Source*: own calculations based on WVS/EVS.

Share of blood donations to family. Blood donations to family members as a fraction of total blood donations; average for 2011–2013. *Source*: Schulz et al. (2019) based on the original data from the WHO Global Status Report on Blood Safety and Availability (2016).

Migrant acceptance index. Gallup's migrant acceptance index is based on three questions. Respondents are asked whether the following situations are "good things" or "bad things": immigrants living in their country, an immigrant becoming their neighbor and immigrants marrying into their families. "A good thing" response is worth three points in the index calculation, a volunteered response of "it depends" or "dont know" is worth one point, and "a bad thing" is worth zero points. The index is a sum of the points across the three questions. The higher the score, the more accepting the population is of migrants. *Source*: Gallup World Poll, 2016–2017.

Social relations, anxiety, and worldview

Generalized trust. Share of respondents replying that "people can be trusted" in the generalized trust question: "Generally speaking would you say that most people can be trusted or that you cant be too careful in dealing with people?" Averages across available years. *Source*: own calculations based on Pew Research Center surveys, WVS/EVS, and Gallup World Poll (as recorded in the 2019 World Happiness Report database).

Trust in neighbors. Based on the following survey question: "How much do you trust the people in your neighborhood?" Possible answers are: a lot (4), some (3), not much (2), not at all (1); data for the year 2018. *Source*: own calculations based on the Gallup World Poll.

Out-group trust. See the definition of the "in- vs. out-group trust" variable above.

Trusted business partner. The share of respondents who believe they can find someone outside their own family to be a trusted business partner; average across available years. *Source*: own calculations based on the Gallup World Poll.

Generalized fairness. Based on the WVS/EVS question: "Do you think that most people would try to take advantage of you if they got the chance, or would they try to be fair?" Answers range on a 0–10 scale, from "most people would try to take advantage of me" (0) to "most people would try to be fair" (10); average across available years 1981–2020. *Source*: own calculations based on WVS and EVS.

Importance of friends and leisure. Based on the WVS/EVS question on how important friends and leisure are in respondents' lives. Answers range on a 1–4 scale, from "not important at all" (1) to "very important' (4); average across available years 1981–2020. *Source*: own calculations based on WVS and EVS.

Blood donations. Voluntary blood donations to non-family per 1,000 inhabitants; average for 2011–2013. *Source*: Schulz et al. (2019) based on the original data from the WHO Global Status Report on Blood Safety and Availability (2016).

Charitable giving. The share of survey respondents who claimed they donated money to a charity in the past month; average across available years. *Source*: own calculations based on the Gallup World Poll.

Helped a stranger. The share of survey respondents who claimed they helped a stranger or someone they didn't know who needed help; average across available years. *Source*: own calculations based on the Gallup World Poll.

Life satisfaction. Average life satisfaction score based on the Cantril life ladder question: "Please imagine a ladder, with steps numbered from 0 at the bottom to 10 at the top. The top of the ladder represents the best possible life for you and the bottom of the ladder represents the worst possible life for you. On which step of the ladder would you say you personally feel you stand at this time?"; average across 2008–2017. *Source*: own calculations based on the World Happiness Report (2019) database which in turn relies on the Gallup World Poll data.

Subjective health. Based on the following WVS/EVS question: "All in all, how would you describe your state of health these days?" Answers coded on a 1–5 ordinal scale from "very poor" (1) to "very good" (5); average across available years 1981–2020. *Source*: own calculations based on WVS/EVS.

Positive affect. Average of three positive affect measures in the Gallup World Poll capturing recent experiences of happiness, smiling/laughing, and enjoyment (on the day before survey date); average across 2008–2017. *Source*: own calculations based on the World Happiness Report (2019) which relies on the Gallup World Poll data.

Negative affect. Average of three negative affect measures in the Gallup World Poll capturing recent experiences of worry, sadness, and anger (on the day before survey date); average across 2008–2017. *Source*: own calculations based on the World Happiness Report (2019) which relies on the Gallup World Poll data.

Control over life. Based on the following question: "Some people feel they have completely free choice and control over their lives, and other people feel that what they do has no real effect on what happens to them. Please use the scale to indicate how much freedom of choice and control you feel you have over the way your life turns out?" Answers coded on a 1–10 ordinal scale from "none at all" (1) to "a great deal" (10); average across available years 1981–2020. *Source*: own calculations based on WVS/EVS.

Freedom of life choices. Fraction of respondents replying "satisfied" to the following question: "Are you satisfied or dissatisfied with your freedom to choose what you do with your life?"; average across 2008–2017. *Source*: own calculations based on the World Happiness Report (2019) which relies on the Gallup World Poll data.

Fatalism. Fraction of respondents claiming to believe in "fate, the idea that the course of your life is largely or wholly preordained." *Source*: own calculations based on the Pew Research Center surveys.

Self-efficacy. Fraction of respondents replying "yes" to the following question: "Can people in this country get ahead by working hard, or not?"; average across available years. *Source*: own calculations based on the Gallup World Poll data.

Zero-sum worldview. A scale constructed to capture a "belief system about the antagonistic nature of social relations – that one person's gain is possible only at the expense of other persons." *Source*: Różycka-Tran et al. (2015; 2018; 2019).

Image of limited good. Based on the WVS/EVS "wealth accumulation" scale varying from "people can only get rich at the expense of others" (1) to "wealth can grow so there's enough for everyone" (10); average across available years 1981–2020. *Source*: own calculations based on WVS/EVS.

Innovation and economic development

Entrepreneurial risk taking. Based on the survey question in the Executive Opinion Survey of the World Economic Forum: "In your country, to what extent do people have an appetite for entrepreneurial risk?" Possible answers ranged on a 1–7 ordinal scale from "not at all" (1) to "to a great extent" (7); 2017–2018 average or most recent period available. *Source*: World Economic Forum, Global Competitiveness Report (2018).

Embracing disruptive ideas. Based on the survey question in the Executive Opinion Survey of the World Economic Forum: "In your country, to what extent do companies embrace risky or disruptive business ideas?" Possible answers ranged on a 1–7 ordinal scale from "not at all" (1) to "to a great extent" (7); 2017–2018 average or most recent period available. *Source*: World Economic Forum, Global Competitiveness Report (2018).

Patent applications. Total number of patent family applications per million population; 2012–2014 average. Computed as the sum of the patent family applications filed in at least two of the major five offices in the World: the European Patent Office, the Japan Patent Office, the Korean Intellectual Property Office, the State Intellectual Property Office of the People's Republic of China, and the United States Patent and Trademark Office. A log transformation is applied to the raw score before it is normalized to a 0 to 100 scale. *Source*: World Economic Forum, Global Competitiveness Report (2018), based on the original data from OECD.

H-index. An index measuring the number of publications and their citations; 2015–2017 average. The H-index measures the number of published papers cited in other papers at least H times. A log transformation is applied to the raw score before it is normalized to a 0 to 100 scale. *Source*: World Economic Forum, Global Competitiveness Report (2018), based on the original data from SCImago.

R&D expenditures. Expenditures on research and development (including basic research, applied research, and experimental development), expressed as a percentage of GDP; data for the year 2015. *Source*: World Economic Forum, Global Competitiveness Report (2018), based on the original data from the UNESCO Institute for Statistics.

Real GDP per capita. Natural logarithm of real gross domestic product per capita measured at purchasing power parity in 2017 international dollars; average across 2008–2017. *Source*: Penn World Table 10.0 and World Development Indicators for Venezuela.

Poverty rate. Natural logarithm of the poverty headcount ratio measured as the percentage of population living on less than $3.20 a day at 2011 puchasing power parity exchange rates; 2008–2017 average. *Source*: own calculations based on the World Development Indicators database.

Life expectancy. Life expectancy at birth, in years; 2008–2017 average. *Source*: own calculations based on the World Development Indicators database.

Mean years of schooling. Average number of completed years of education of a country's population aged 25 years and older, excluding years spent repeating individual grades; data for 2015. *Source*: World Economic Forum, Global Competitiveness Report (2018), based on the original data from UNESCO and the Wittgenstein Centre for Demography and Global Human Capital.

Human development index. Human development index; average across 2010, 2014, 2015, 2017. *Source*: UNDP Human Development Report (2020) database.

Exposure to misfortunes

Exposure to natural disasters. Share of population physical exposed to earthquakes, storms, floods, droughts, and sea-level rise. *Source*: WorldRiskReport (2020).

Exposure to agricultural drought. An index of exposure to agricultural drought based on historical climate conditions. *Source*: Meza et al. (2020).

Pathogen richness. The number of all infectious diseases listed for a given country in the Global Infectious Disease and Epidemiology Network; April–August 2007. *Source*: Fincher and Thornhill (2008).

Armed civil conflict. The natural logarithm of one plus the number of new civil conflict onsets per year during the 1960–2017 time period, based on the UCDP/PRIO armed conflict dataset. *Source*: Arbatlı et al. (2020).

Unemployment rate. Modeled ILO estimate of the unemployment rate; 2008–2017 average. *Source*: own calculations based on the World Development Indicators database.

B Additional analyses

Table B.1: Socio-demographic correlates: linear probability model

	(1)	(2)	(3)	(4)	(5)	(6)	(7)	(8)
**Age**	−0.002	−0.005**	−0.007***	−0.005*	−0.006*	−0.007***	−0.007***	−0.006**
	(0.002)	(0.002)	(0.002)	(0.003)	(0.003)	(0.002)	(0.002)	(0.003)
**Gender**: woman	0.043***	0.041***	0.014**	0.010	0.010	0.035***	0.038***	0.010
	(0.008)	(0.008)	(0.006)	(0.007)	(0.007)	(0.007)	(0.007)	(0.007)
**Education**: vs. "primary or less"								
Some or completed secondary		−0.034***	−0.031***	−0.030***	−0.031***	−0.029***	−0.040***	−0.028***
		(0.008)	(0.008)	(0.009)	(0.009)	(0.007)	(0.007)	(0.008)
Above secondary		−0.070***	−0.062***	−0.063***	−0.065***	−0.061***	−0.076***	−0.060***
		(0.012)	(0.013)	(0.015)	(0.015)	(0.011)	(0.011)	(0.014)
**Econ. situation**: vs. "very bad"								
Somewhat bad			−0.031***	−0.029***	−0.029***			−0.029***
			(0.008)	(0.009)	(0.009)			(0.009)
Somewhat good			−0.064***	−0.052***	−0.052***			−0.053***
			(0.009)	(0.009)	(0.009)			(0.009)
Very good			−0.064***	−0.060***	−0.060***			−0.058***
			(0.011)	(0.013)	(0.013)			(0.013)
**Household size**: vs. 1–3								
4–5				0.004	0.004			0.003
				(0.005)	(0.005)			(0.005)
6 and above				0.017**	0.017**			0.016**
				(0.008)	(0.008)			(0.007)
**Urban resident**					0.009			0.011
					(0.008)			(0.009)
**Religion**: vs. Christian								
Muslim						0.016	0.016	−0.007
						(0.022)	(0.022)	(0.026)
Unaffiliated						−0.041***	−0.022*	0.018
						(0.013)	(0.011)	(0.022)
**Imp. of religion**: vs. "not at all"								
Not too important						0.066***		0.056**
						(0.010)		(0.026)
Somewhat important						0.143***		0.104***
						(0.011)		(0.028)
Very important						0.149***		0.104***
						(0.013)		(0.028)
**Belief in god**							0.175***	
							(0.015)	
Observations	135,693	133,244	101,264	75,746	75,746	129,037	101,556	73,849
Countries	95	94	74	58	58	94	73	58

Notes. The binary dependent variable is personal belief in witchcraft. Ordinary least-squares estimates from the linear probability regressions are reported in all columns. Standard errors clustered by country are shown in parentheses. ***, **, and * denote statistical significance at the 1, 5, and 10 percent level, respectively. Country fixed effects are included in all specifications. Age is measured in tens of years. The number of observations and countries for each specification reflects data availability constraints.

Table B.2: Socio-demographic correlates: accounting for wave fixed effects

	(1)	(2)	(3)	(4)	(5)	(6)	(7)	(8)
**Age**	-0.008^{**}	-0.012^{***}	-0.010^{***}	-0.003	-0.004	-0.013^{***}	-0.011^{***}	-0.004
	(0.004)	(0.004)	(0.004)	(0.004)	(0.004)	(0.004)	(0.003)	(0.004)
**Gender**: woman	0.044^{***}	0.042^{***}	0.016^{*}	0.008	0.007	0.037^{***}	0.042^{***}	0.009
	(0.009)	(0.009)	(0.008)	(0.008)	(0.008)	(0.009)	(0.009)	(0.008)
**Education**: vs. "primary or less"								
Some or completed secondary		-0.059^{***}	-0.041^{**}	-0.037^{*}	-0.045^{**}	-0.048^{***}	-0.053^{***}	-0.039^{**}
		(0.019)	(0.018)	(0.020)	(0.019)	(0.018)	(0.020)	(0.018)
Above secondary		-0.091^{***}	-0.069^{***}	-0.056^{**}	-0.068^{***}	-0.073^{***}	-0.083^{***}	-0.061^{**}
		(0.026)	(0.023)	(0.027)	(0.026)	(0.024)	(0.022)	(0.026)
**Econ. situation**: vs. "very bad"								
Somewhat bad			-0.043^{***}	-0.044^{***}	-0.045^{***}			-0.046^{***}
			(0.012)	(0.013)	(0.013)			(0.013)
Somewhat good			-0.097^{***}	-0.081^{***}	-0.081^{***}			-0.084^{***}
			(0.016)	(0.017)	(0.017)			(0.017)
Very good			-0.102^{***}	-0.090^{***}	-0.091^{***}			-0.091^{***}
			(0.020)	(0.022)	(0.022)			(0.022)
**Household size**: vs. 1–3								
4–5				0.010	0.012			0.012
				(0.008)	(0.008)			(0.008)
6 and above				0.039^{**}	0.042^{***}			0.039^{***}
				(0.016)	(0.015)			(0.015)
**Urban resident**					0.047^{***}			0.048^{***}
					(0.014)			(0.014)
**Religion**: vs. Christian								
Muslim						0.060	0.042	0.027
						(0.037)	(0.034)	(0.045)
Unaffiliated						-0.069^{***}	-0.018	0.036
						(0.024)	(0.019)	(0.027)
**Imp. of religion**: vs. "not at all"								
Not too important						0.117^{***}		0.057^{**}
						(0.015)		(0.027)
Somewhat important						0.197^{***}		0.092^{***}
						(0.018)		(0.028)
Very important						0.194^{***}		0.087^{***}
						(0.022)		(0.029)
**Belief in god**							0.245^{***}	
							(0.017)	
Observations	135,693	133,244	101,264	75,746	75,746	129,037	101,556	73,849
Countries	95	94	74	58	58	94	73	58

Notes. The binary dependent variable is personal belief in witchcraft. Maximum likelihood estimates of marginal effects from probit regressions are reported in all columns. Standard errors clustered by country are shown in parentheses. ***, **, and * denote statistical significance at the 1, 5, and 10 percent level, respectively. Survey wave fixed effects are included in all specifications (the waves correspond to those reported in table A.1, with the U.S. incorporated into the 2008-2009 wave based on the survey year). Age is measured in tens of years. The number of observations and countries for each specification reflects data availability constraints.

C Cross-country patterns in scatterplots

This section further illustrates selected cross-country patterns from the main text of the paper. With the exception of quadratic relationships for development indicators in figure C.6, represented by augmented component-plus-residual plots, all panels are standard scatterplots of residuals after accounting for continental fixed effects. The reported t-statistics are based on heteroskedasticity-robust standard errors.

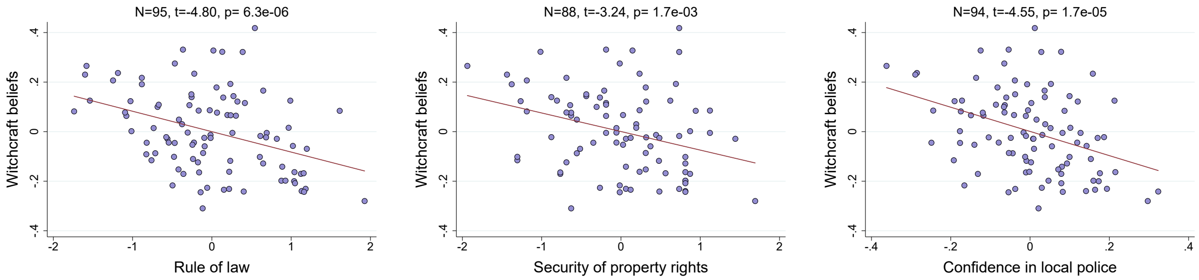

Figure C.1: Witchcraft beliefs and institutions.

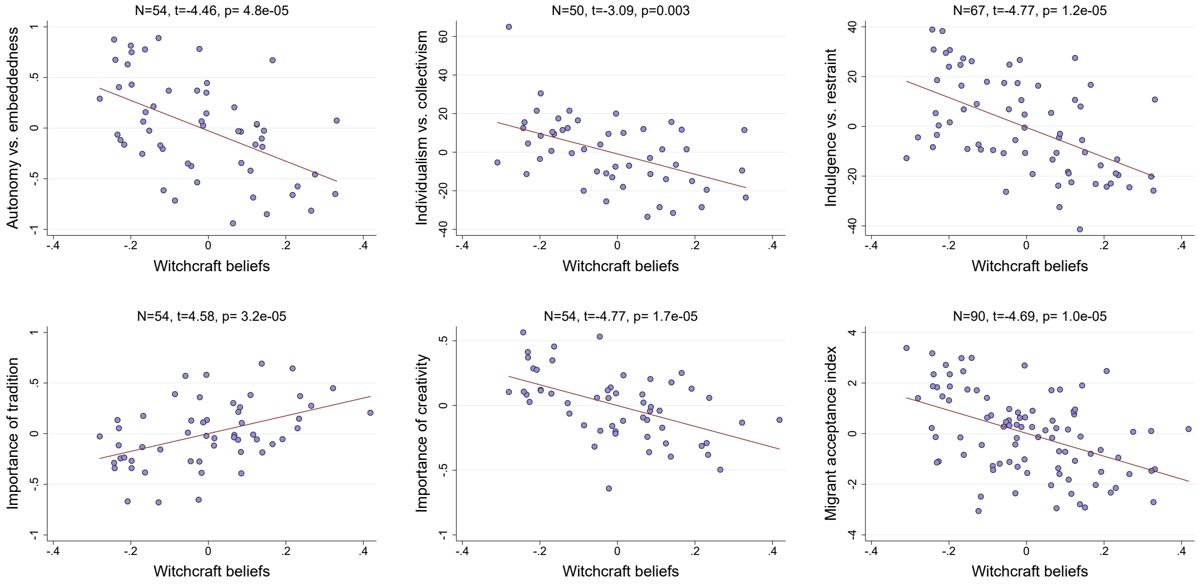

Figure C.2: Witchcraft beliefs, conformity, and in-group bias.

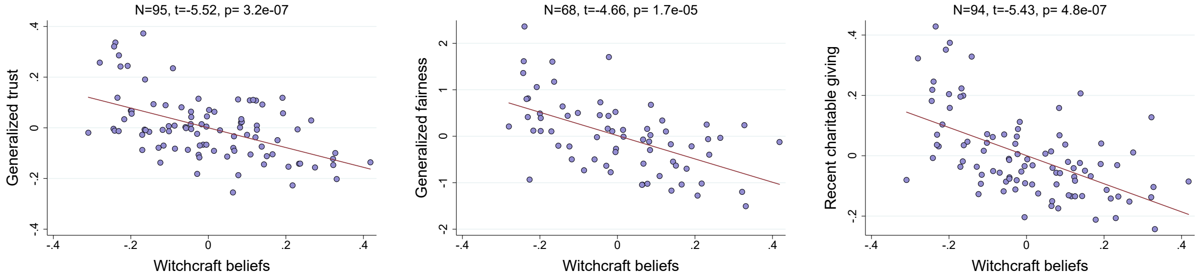

Figure C.3: Witchcraft beliefs and ruptured social relations.

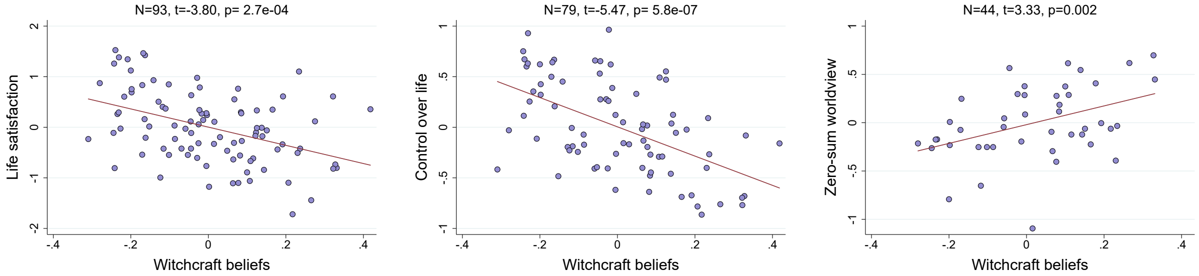

Figure C.4: Witchcraft beliefs, anxiety, and worldview.

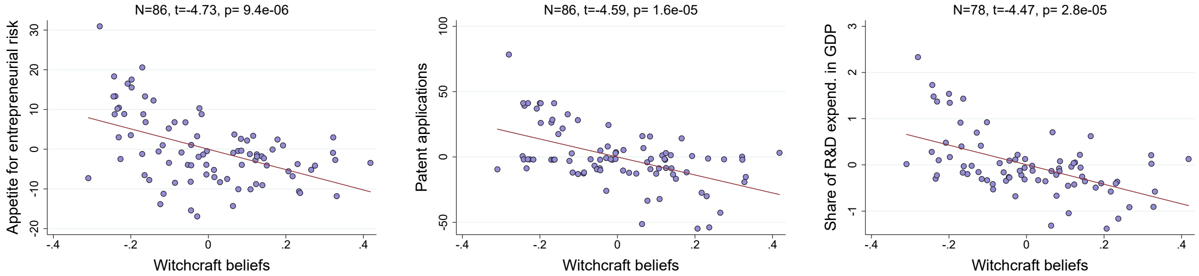

Figure C.5: Witchcraft beliefs and innovation.

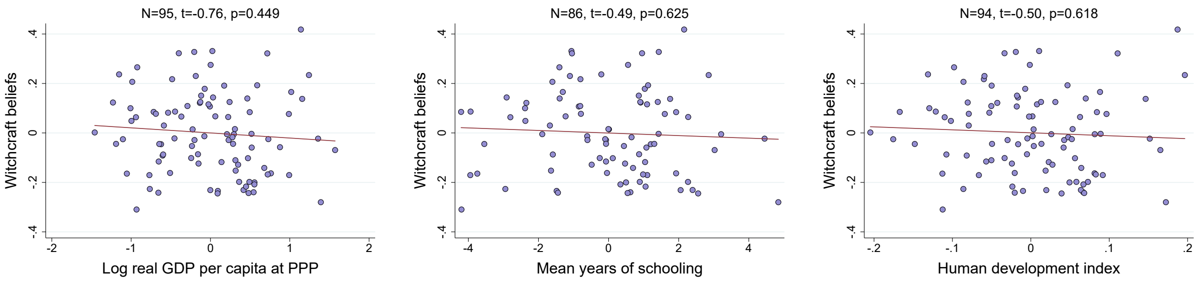

(a) Linear relationship

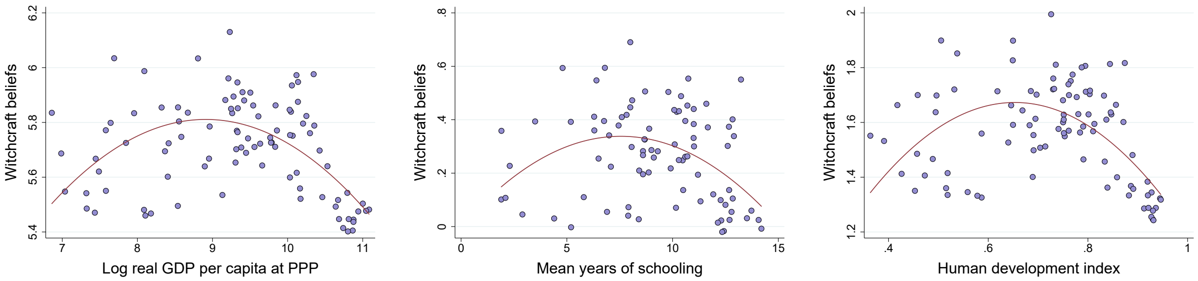

(b) Quadratic relationship

Figure C.6: Witchcraft beliefs and development.

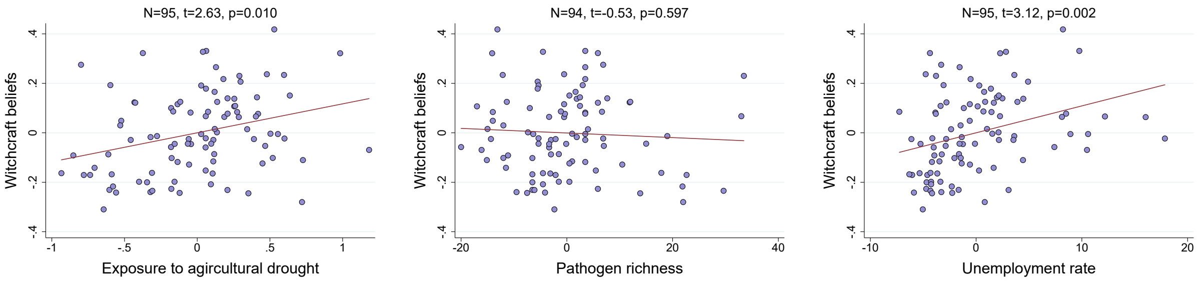

Figure C.7: Witchcraft beliefs and exposure to misfortunes.

References

Arbatlı, Cemal Eren, Quamrul H. Ashraf, Oded Galor, and Marc Klemp, "Diversity and Conflict," *Econometrica*, 2020, *88* (2), 727–797.

Fincher, Corey L. and Randy Thornhill, "Assortative Sociality, Limited Dispersal, Infectious Disease and the Genesis of the Global Pattern of Religion Diversity," *Proceedings of the Royal Society B: Biological Sciences*, 2008, *275* (1651), 2587–2594.

Galor, Oded and Ömer Özak, "The Agricultural Origins of Time Preference," *American Economic Review*, October 2016, *106* (10), 3064–3103.

Hofstede, Geert, Gert Jan Hofstede, and Michael Minkov, *Cultures and Organizations: Software of the Mind*, 3rd ed., New York: McGraw-Hill, 2010.

Meza, I., S. Siebert, P. Döll, J. Kusche, C. Herbert, E. Eyshi Rezaei, H. Nouri, H. Gerdener, E. Popat, J. Frischen, G. Naumann, J. V. Vogt, Y. Walz, Z. Sebesvari, and M. Hagenlocher, "Global-Scale Drought Risk Assessment for Agricultural Systems," *Natural Hazards and Earth System Sciences*, 2020, *20* (2), 695–712.

Nunn, Nathan and Diego Puga, "Ruggedness: The Blessing of Bad Geography in Africa," *Review of Economics and Statistics*, February 2012, *94* (1), 20–36.

Różycka-Tran, Joanna, Guido Alessandri, Paweł Jurek, and Michał Olech, "A Test of Construct Isomorphism of the Belief in a Zero-Sum Game Scale: A Multilevel 43-Nation Study," *PLoS ONE*, September 2018, *13* (9), 1–15.

Różycka-Tran, Joanna, Jarosław P. Piotrowski, Magdalena Żemojtel-Piotrowska, Paweł Jurek, Evgeny N. Osin, Byron G. Adams, Rahkman Ardi, Sergiu Bălătescu, Arbinda Lal Bhomi, Sergey A. Bogomaz, Jan Cieciuch, Amanda Clinton, Gisela T. de Clunie, and Anna Z. Czarna et al., "Belief in a Zero-Sum Game and Subjective Well-Being across 35 Countries," *Current Psychology*, May 2019.

Różycka-Tran, Joanna, Paweł Boski, and Bogdan Wojciszke, "Belief in a Zero-Sum Game as a Social Axiom: A 37-Nation Study," *Journal of Cross-Cultural Psychology*, May 2015, *46* (4), 525–548.

Schulz, Jonathan F., Duman Bahrami-Rad, Jonathan P. Beauchamp, and Joseph Henrich, "The Church, Intensive Kinship, and Global Psychological Variation," *Science*, November 2019, *366* (6466), eaau5141.

Schwartz, Shalom H., "National Culture as Value Orientations: Consequences of Value Differences and Cultural Distance," in Victor A. Ginsburgh and David Throsby, eds., *Handbook of the Economics of Art and Culture*, Vol. 2, Amsterdam: Elsevier, 2014, chapter 20, pp. 547–586.

Uz, Irem, "The Index of Cultural Tightness and Looseness Among 68 Countries," *Journal of Cross-Cultural Psychology*, 2015, *46* (3), 319–335.
